# Independent and combined associations of high-density lipoprotein cholesterol-modified triglyceride-glucose index with all-cause and cardiovascular mortality in patients with acute decompensated heart failure

**DOI:** 10.3389/fendo.2025.1629066

**Published:** 2025-07-29

**Authors:** Shiming He, Lin Xie, Guobo Xie, Guoan Jian, Kun Jiang, Zihao Lu, Shuhua Zhang, Qun Wang, Hengcheng Lu, Zhiyu Xiong, Zhiting Wu, Guotai Sheng, Hengli Lai, Wei Wang, Yang Zou

**Affiliations:** ^1^ Jiangxi Medical College, Nanchang University, Nanchang, Jiangxi, China; ^2^ Jiangxi Cardiovascular Research Institute, Jiangxi Provincial People’s Hospital, The First Affiliated Hospital of Nanchang Medical College, Nanchang, Jiangxi, China; ^3^ Department of Cardiology, Jiangxi Provincial People’s Hospital, The First Affiliated Hospital of Nanchang Medical College, Nanchang, Jiangxi, China

**Keywords:** acute decompensated heart failure, TyG/HDL-C ratio, insulin resistance, all-cause mortality, cardiovascular mortality acute decompensated heart failure, cardiovascular mortality

## Abstract

**Introduction:**

Dysregulation of glucolipid metabolism is a central pathological mechanism underlying acute decompensated heart failure (ADHF) and significantly impacts its poor prognosis. This study aims to investigate the association between the high-density lipoprotein cholesterol-modified triglyceride-glucose index (defined as TyG/HDL-C) and their interaction with 30-day mortality in patients with ADHF.

**Methods:**

From 2018 to 2024, 2,329 ADHF patients enrolled in the Jiangxi-ADHF II cohort were included. Multivariable Cox regression models were utilized to evaluate the association between TyG/HDL-C ratio and 30-day all-cause/cardiovascular mortality risk. A 3-dimensional interaction model was employed to examine the dose-response relationships of TyG and HDL-C with mortality risk. Additionally, exploratory mediation models were constructed to investigate potential mediating effects of inflammation, oxidative stress, and nutritional metabolism in the association between TyG/HDL-C ratio and mortality risk.

**Results:**

At 30-day follow-up, 150 deaths occurred, 115 of which were cardiovascular. Multivariable Cox regression showed that each standard deviation increase in TyG/HDL-C ratio increased 30-day all-cause mortality by 24% and cardiovascular mortality by 20%. These findings demonstrated robustness across sensitivity analyses conducted from four dimensions: model adjustment, causal timing, population heterogeneity, and data integrity. Notably, the subsequent 3-dimensional interaction model analysis revealed a complex U-shaped association — resembling a concave surface of a radio telescope — between the combined effects of TyG index and HDL-C on mortality risk. Specifically, both excessively low and high combinations of TyG index and HDL-C were associated with elevated 30-day mortality risk in ADHF patients, while the lowest mortality risk interval occurred when the TyG index remained within 7.5–9.0 and HDL-C levels were maintained at 1.0–1.5 mmol/L. Mediation analysis further suggested that inflammatory and nutritional pathways might serve as significant mediators of mortality risk related to TyG/HDL-C ratio.

**Discussion:**

The TyG/HDL-C ratio emerged as an independent predictor of short-term all-cause and cardiovascular mortality in ADHF patients, demonstrating significant enhancement in predictive performance for these outcomes. Most notably, the concave-shaped interaction pattern revealed by 3-dimensional interaction analysis provided an evidence-based threshold framework for metabolic management in ADHF patients, which may hold substantial clinical significance for reducing future mortality risks in this population.

## Graphical Abstract

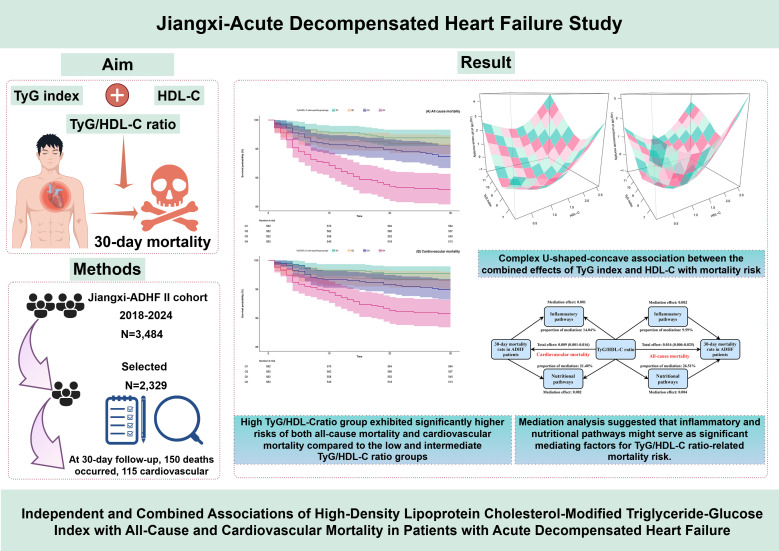

## Background

Heart failure (HF) represents a cardiovascular syndrome characterized by a chronic clinical course, accompanied by a significant symptom burden and high mortality rates (1, 2). Triggered by factors such as infection, ischemia, or volume overload, disruption of chronic compensatory mechanisms can rapidly precipitate acute decompensated HF(ADHF), a condition pathologically defined by volume overload and hemodynamic disturbances (3). Epidemiological evidence reveals a high incidence of short-term adverse outcomes in ADHF patients, with 30-day readmission and all-cause mortality rates approximating 25% and 10%, respectively (4, 5). Of particular concern is the unequal distribution of regional healthcare resources, which disproportionately increases the disease burden in low- and middle-income countries (6). Exemplified by China, the interplay between accelerated population aging and the rising prevalence of metabolic disorders—such as diabetes and obesity—is fueling a sharp increase in HF incidence (7). Additionally, factors such as inadequate chronic HF management and dietary/environmental exposures, among other factors, may further increase the risk of ADHF occurrence (8, 9). Therefore, establishing an early precision risk stratification system based on multidimensional biomarkers has significant public health implications for optimizing clinical decision-making pathways and reducing ADHF mortality rates.

In recent years, the driving role of metabolic disorders in HF progression has attracted significant attention among healthcare professionals. Insulin resistance (IR), recognized as the core pathological basis of metabolic syndrome, not only directly disrupts cardiomyocyte energy metabolism via glucolipotoxicity but also triggers systemic inflammatory cascades, thereby exacerbating cardiorenal crosstalk injury in ADHF patients (10–12). Notably, the triglyceride-glucose (TyG) index has gained prominence due to its efficient representation of IR (13). Compared with the hyperinsulinemic-euglycemic clamp technique, the TyG index demonstrates comparable diagnostic accuracy while offering advantages such as low cost, high accessibility, and non-invasiveness (13). Moreover, it has been consistently validated as an independent predictor of mortality risk across multiple HF subtypes (14–20). However, exclusive reliance on the TyG index or conventional lipid metrics may result in misinterpretation of metabolic dysregulation holistically. For instance, triglycerides (TG) primarily reflect the metabolic status of very low-density lipoproteins but overlook critical pathological aspects of high-density lipoprotein cholesterol (HDL-C) functional deficits (21). Evidence indicates that HDL-C functional depletion exacerbates ADHF progression through cardiomyocyte lipid deposition and oxidative stress (22). These findings collectively highlight a central issue: the impact of lipid metabolic dysregulation on ADHF prognosis requires systematic evaluation from dual dimensions—”lipid burden” (represented by the TyG index) and “anti-inflammatory defense” (characterized by HDL function). The limitations of single indicators make it challenging to fully capture the interactive effects of metabolic imbalances in ADHF patients. To address this core challenge, Gao et al. recently proposed that the ratio of TyG index to HDL-C (TyG/HDL-C) demonstrated superior metabolic advantages through their novel combined metric. Their study reported a diagnostic accuracy of 92.9% for metabolic dysfunction-associated fatty liver disease using this TyG/HDL-C ratio (23). Subsequently, Tong et al. confirmed its clinical applicability by demonstrating the ratio’s efficacy in assessing coronary artery calcification risk, thereby significantly expanding the evidence base for its application in cardiovascular diseases (24). The current study focuses on ADHF, a severe cardiovascular condition, and employs a retrospective cohort study design to investigate the independent association between the TyG/HDL-C ratio and both all-cause mortality and cardiovascular-specific mortality in ADHF patients, aiming to provide evidence-based support for the establishment of a biomarker-driven precision prognostic evaluation framework.

## Methods

### Study population

The Jiangxi-ADHF II study is a retrospective cohort study designed to integrate clinical data from ADHF patients admitted to Jiangxi Provincial People’s Hospital, aiming to construct a regional high-quality cohort for optimizing early risk stratification and prognostic management. This study consecutively enrolled 3,484 ADHF patients admitted to Jiangxi Provincial People’s Hospital between January 2018 and January 2024. ADHF diagnoses were established according to the latest HF guidelines available at the time from the European Society of Cardiology and the American College of Cardiology/American Heart Association. The primary diagnostic criteria encompass clinical manifestations and objective examination findings in the following two domains: (1) Symptomatic deterioration (must meet at least one of the following): (a) Dyspnea (including exertional dyspnea, paroxysmal nocturnal dyspnea, or orthopnea); (b) Signs of systemic congestion (including lower extremity edema, hepatic congestion, or ascites); (c) Hypoperfusion manifestations (including oliguria/anuria, cold extremities, altered mental status, hyperlactatemia, or metabolic acidosis). (2) Objective evidence of heart failure (must meet at least one of the following): (a) Signs of pulmonary edema confirmed by physical examination or chest radiography; (b) Elevated natriuretic peptide levels (B-type natriuretic peptide [BNP] or N-terminal pro-BNP [NT-proBNP]); (c) Echocardiographic evidence of cardiac structural and/or functional abnormalities.

**Figure 1:**
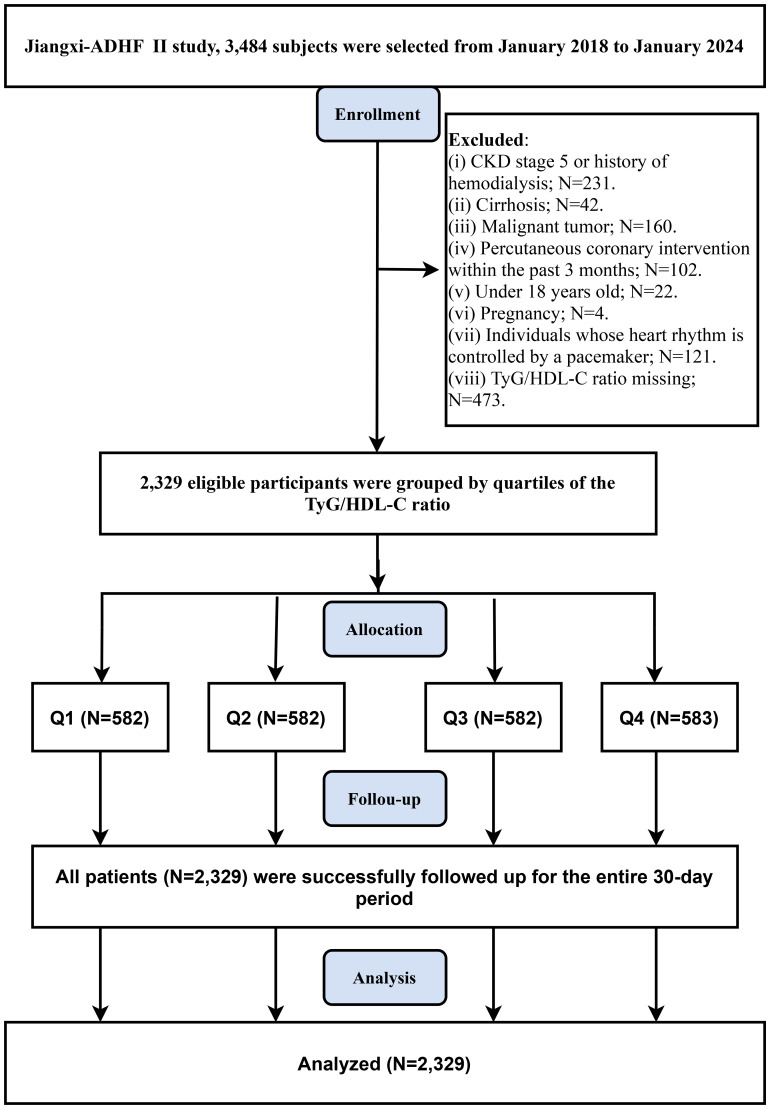
Flow chart for inclusion and exclusion of study participants.

Based on requirements for pathological heterogeneity and data integrity, this study excluded patients with conditions significantly affecting fluid-sodium retention (including uremic patients, those undergoing hemodialysis (N=231), or those with liver cirrhosis [N=42]), patients with cardiac pacemakers (N=121: where autonomous nervous control was expected to be limited), patients who recently underwent interventional procedures (N=102: as reperfusion therapy critically impacts short-term prognosis), patients with concurrent malignancies (n=160), patients with age <18 years (N=22), pregnant patients (N=4), and cases with missing TyG/HDL-C data (N=473). Ultimately, 2,329 patients were included in the analysis. The study flowchart and screening details are provided in **Figure 1**.

### Ethical approval

The implementation of this study was approved by the Ethics Committee of Jiangxi Provincial People’s Hospital (IRB No: 2024-01). Informed consent for the use of study data was obtained from participants and their families. The entire research process was conducted in full compliance with the ethical principles outlined in the Declaration of Helsinki, and the findings were presented adhering to the STROBE guidelines.

### Data collection

The baseline data collection in this study employed a dual-entry and blinded verification quality control system: Two research assistants, trained through standardized protocols, independently recorded demographic characteristics (gender, age) and clinical data, including smoking and drinking status, comorbidities [hypertension, diabetes, stroke, coronary heart disease (CHD)], New York Heart Association (NYHA) functional classification at admission, vital signs [blood pressure (BP)], echocardiographic parameters [left ventricular ejection fraction (LVEF)] and medication information during hospitalization [Includes the use of diuretics, beta-blockers, digitalis, sodium-dependent glucose transporters 2, angiotensin-converting enzyme inhibitors/angiotensin receptor inhibitors/angiotensin receptor neprilysin inhibitors, and vasopressor medications. All data were cross-verified before inclusion in the final analysis. It should be noted that BP measurements were conducted in accordance with the guidelines of the European Society of Hypertension, using an Omron medical automatic sphygmomanometer (HBP-1300). Measurements were taken after patients were admitted and in a resting state (sitting or supine rest at the bedside for ≥5 minutes), with systolic BP (SBP) and diastolic BP (DBP) recorded in mmHg and precise to the nearest whole number. Comorbidity diagnoses were determined based on a multidimensional evidence chain, including patient-reported medical history, pharmacotherapy regimens (e.g., continuous use of antihypertensive/antidiabetic medications), and ≥1 specialist physician diagnoses documented in the electronic medical record system.

All laboratory tests were performed at the Clinical Laboratory Center of Jiangxi Provincial People’s Hospital. Blood samples were collected within 24 hours of admission. Blood biochemical parameters, including albumin (Alb), alanine aminotransferase (ALT), aspartate aminotransferase (AST), gamma-glutamyl transferase (GGT), creatinine (Cr), uric acid (UA), total cholesterol (TC), TG, low-density lipoprotein cholesterol (LD-C), HDL-C, and fasting plasma glucose (FPG), were measured using a HITACHI LAbOSPECT 008 automated analyzer (Hitachi High-Tech Co, Tokyo, Japan). Complete blood count parameters, such as white blood cell count (WBC), red blood cell count (RBC), and platelet count (PLT), were obtained via the Sysmex XN-3000 (Sysmex Co, Kobe, Japan) hematology analyzer. Cardiac biomarker NT-proBNP was quantitatively analyzed using an electrochemiluminescence immunoassay. The Sysmex XN-3000 automated hematology analyzer and Hitachi LABOSPECT 008 automated biochemical analyzer used in this study demonstrate excellent analytical performance. Both instruments feature high precision, optimal linear ranges, reliable clinical reportable ranges and reference intervals, and fully satisfy clinical testing requirements (25, 26). According to manufacturer-provided performance verification data, the coefficients of variation for all tested parameters are controlled within stringent quality control standards: blood count ≤6%; glucose ≤5%; BUN ≤4%; Cr ≤5%; UA ≤4%; lipid profile ≤4%; hepatic enzymes ≤5%; and Alb ≤4%. It must be emphasized that blood samples for FPG, lipid profiles (TC, TG, LDL-C, HDL-C), and liver enzymes (ALT, AST, GGT) were collected under strict fasting conditions—defined as either ≥8 hours of fasting since last meal at admission or additional venous blood sampling the next morning in a fasting state, to exclude dietary metabolic interference.

For research specimens, our laboratory has established a rigorous quality control system: (1) Pre-analysis verification: All samples must undergo testing only after confirming that daily internal quality control results are within acceptable limits, ensuring the detection system operates in a stable state. (2) Dual quality control mechanism: Internal monitoring: Daily internal quality control is performed to evaluate the performance of all detection parameters. External validation: Regular participation in national and provincial external quality assessment programs (5–10 times annually) ensures comparability and reliability of results. (3) Out-of-control management: In the event of assay deviations, the laboratory immediately suspends relevant testing activities. Testing resumes only after thorough root cause analysis, implementation of corrective actions, and confirmation of restored control status.

### Calculation of TyG and TyG/HDL-C ratio


TyG index=ln[TG(mg/dL)×FPG(mg/dL)/2]

TyG/HDL−C ratio=TyG index/HDL−C(mmol/L)

### Study outcomes

This study defined the admission time of ADHF patients as the starting point for follow-up, with a duration of 30 days. The primary outcome was all-cause mortality within 30 days, while the secondary outcome was cardiovascular mortality during the same 30-day follow-up. Survival status of ADHF patients was ascertained by trained medical professionals through multiple methods, including text messaging, telephone follow-ups, and in-person interviews (either in outpatient clinics or inpatient wards).

### Statistical analysis

Data analysis in this study was performed using Free Statistics version 1.7, R language version 3.4.1, and Empower(R) version 2.0 statistical software. Participants were stratified into four groups (Q1-Q4) based on quartiles of the TyG/HDL-C ratio. Baseline characteristics were summarized as frequency (percentage), mean [standard deviation (SD)], or median (interquartile range) according to variable type. Intergroup differences were evaluated using Student’s t-test (for normally distributed variables), one-way analysis of variance (for multi-group comparisons of means), or non-parametric tests (for non-normally distributed/ranked variables). Statistical significance was defined as a two-sided *p*-value <0.05.

A correlation analysis framework was constructed using the Cox proportional hazards model. Variance inflation factors were calculated to exclude covariates with multicollinearity (**Supplementary Table S1**) (27). Kaplan-Meier analysis was employed to generate survival curves stratified by quartiles of the TyG/HDL-C ratio. Three progressively adjusted multivariable models were constructed, and hazard ratios (HRs) per 1-standard deviation (SD) increase were calculated (the TyG/HDL-C ratio was incorporated into the models after Z-score standardization): Model I adjusted for demographic characteristics of ADHF patients (gender, age, drinking, and smoking status). Model II additionally adjusted for clinical comorbidities (hypertension, diabetes, stroke, CHD) and potential influence of LVEF. Model III, serving as the final model, further extensively adjusted for hematologic indices (WBC, RBC, PLT, AST, GGT, Alb, Cr, BUN, UA, TC, LDL, NT-proBNP). Based on the final model, we also employed restricted cubic splines (RCS) fitting and visualization to analyze the dose-response relationship between TyG/HDL-C ratio and 30-day all-cause and cardiovascular mortality in ADHF patients. To further elucidate the important contribution of the TyG/HDL-C ratio to 30-day mortality, we additionally employed OpenGL technology to generate a 3-dimensional (3D) surface plot visualizing the joint association of TyG index, HDL-C, and 30-day mortality, with covariate adjustments consistent with Model III (28, 29). After fitting the Cox regression models, schoenfeld residuals were used to test the proportional hazards assumption for covariates included in the models (**Supplementary Tables S2**, **S3**), and the results indicated that all covariates met the proportional hazards assumption (all-cause mortality: Global Schoenfeld Test *P*=0.053; cardiovascular mortality: Global Schoenfeld Test *P*=0.857). In analyses with all-cause mortality as the outcome, AST and PLT demonstrated marginal violations of the assumption (*P*=0.03 and *P*=0.01, respectively); however, their Schoenfeld residual plots did not exhibit evident time-dependent trends (**Supplementary Figure S1**), and these variables were retained in the models accordingly.

For predictive performance evaluation, we utilized receiver operating characteristic curve analysis to calculate the area under the curve (AUC), C-index, best threshold, sensitivity, and specificity of the TyG/HDL-C ratio and TyG index for predicting all-cause and cardiovascular mortality. Differences in AUC values were compared using DeLong’s test. Furthermore, we investigated whether adding the TyG/HDL-C ratio to the existing biomarker NT-proBNP could improve the predictive performance for 30-day mortality, and calculated the C-index and Net Reclassification Improvement to quantify its ability to enhance the predictive capacity of the NT-proBNP-based model.

To evaluate the generalizability of our findings to broader populations, we conducted subgroup analyses stratified by mean age (<69 vs. ≥69 years), gender (male vs. female), LVEF (<50% vs. ≥50%), NYHA classification (III vs. IV), and comorbidity status [hypertension (yes/no), diabetes (yes/no), stroke (yes/no), and CHD (yes/no)]. Likelihood ratio tests were used to assess heterogeneity across subgroups and determine the presence of interaction effects.

Exploratory mediation analysis was performed to assess whether several common pathways mediated the association between the TyG/HDL-C ratio and mortality. Based on the bootstrap method, we quantified the mediation effects of oxidative stress (GGT) (30), inflammation (WBC) (31), and nutritional status (Alb) (32) on this association. The mediation contribution was expressed as the ratio of indirect effect to total effect (33).

### Sensitivity analyses

1. In the initial analysis, BP parameters, TG, and FPG were not included in the multivariate model due to potential collinearity concerns between BP measurements and hypertension, as well as possible multicollinearity between the TyG/HDL-C ratio and its components. However, collinearity diagnostics based on variance inflation factors demonstrated no significant multicollinearity between SBP, DBP, TG, FPG and other covariates (**Supplementary Table S1**). Consequently, we additionally adjusted for SBP, DBP, TG and FPG in Model III for sensitivity analysis.
2. Exclusion of patients who died within 48 hours after admission to minimize potential reverse causation.
3. Removal of subgroups with concurrent hypertension, diabetes, stroke, and CHD.
4. Re-running primary analyses after handling missing data (**Supplementary Table S4**) using multiple imputations.
5. Pharmacotherapy is a significant determinant of short-term prognosis. In sensitivity analyses, we further adjusted for HF medications administered during hospitalization, including beta-blockers, diuretics, angiotensin-converting enzyme inhibitors/angiotensin receptor blockers/angiotensin receptor neprilysin inhibitors, digitalis, sodium-glucose cotransporter 2 inhibitors, and vasopressor medications.
6. To further explore whether the short-term prognosis of ADHF exhibits significant time-dependent fluctuations during short-term follow-up, we examined the associations between the TyG/HDL-C ratio and mortality outcomes at 20-day and 25-day follow-up intervals.
7. To test the robustness of the 3D interaction results, we constructed a heatmap visualizing the associations among the TyG index, HDL-C, and 30-day mortality in ADHF patients, providing an intuitive demonstration of potential thresholds for the joint association of the TyG index and HDL-C with 30-day mortality outcomes.

## Results

### Baseline characteristics of the study population stratified by TyG/HDL-C ratio

A total of 2,329 eligible ADHF patients were enrolled in this study, including 58.74% males (n=1,368) and 41.26% females (n=961), with a mean age of 69 years. Stratification by TyG/HDL-C ratio quartiles (**Table 1**) showed significant differences in baseline characteristics between Q4 (11.00 to 70.44) and Q1 (3.07 to 7.14) patients: Q4 patients were younger, more frequently male, had higher diabetes prevalence, and exhibited more severe cardiac dysfunction. Laboratory findings revealed elevated WBC, RBC, PLT, ALT, AST, GGT, Cr, BUN, UA, TG, FPG, and NT-proBNP, but lower Alb, TC, and HDL-C in Q4 patients, alongside worsened hemodynamics (lower SBP) and impaired systolic function.

**Table 1:** Summary of baseline characteristics of the study population according to TyG/HDL-C ratio quartiles group.

Variable	TyG/HDL-C ratio quartiles				*P*-value
	Q1 (3.07-7.14)	Q2 (7.14-8.75)	Q3 (8.76-10.99)	Q4 (11.00-70.44)	
No. of subjects	582	582	582	583	
Age (years)	74.00 (65.00-81.00)	71.00 (63.00-80.00)	70.00 (59.00-78.00)	68.00 (57.00-77.00)	<0.001
Gender					<0.001
Male	292 (50.17%)	345 (59.28%)	343 (58.93%)	388 (66.55%)	
Female	290 (49.83%)	237 (40.72%)	239 (41.07%)	195 (33.45%)	
Hypertension (n,%)					0.583
No	325 (55.84%)	309 (53.09%)	313 (53.78%)	330 (56.60%)	
Yes	257 (44.16%)	273 (46.91%)	269 (46.22%)	253 (43.40%)	
Diabetes (n,%)					<0.001
No	489 (84.02%)	448 (76.98%)	397 (68.21%)	369 (63.29%)	
Yes	93 (15.98%)	134 (23.02%)	185 (31.79%)	214 (36.71%)	
Stroke (n,%)					0.488
No	470 (80.76%)	482 (82.82%)	489 (84.02%)	486 (83.36%)	
Yes	112 (19.24%)	100 (17.18%)	93 (15.98%)	97 (16.64%)	
CHD (n,%)					0.259
No	398 (68.38%)	398 (68.38%)	385 (66.15%)	371 (63.64%)	
Yes	184 (31.62%)	184 (31.62%)	197 (33.85%)	212 (36.36%)	
NYHA classification (n,%)					<0.001
III	425 (73.02%)	406 (69.76%)	394 (67.70%)	351 (60.21%)	
IV	157 (26.98%)	176 (30.24%)	188 (32.30%)	232 (39.79%)	
Drinking status					0.673
No	519 (89.18%)	525 (90.21%)	522 (89.69%)	532 (91.25%)	
Yes	63 (10.82%)	57 (9.79%)	60 (10.31%)	51 (8.75%)	
Smoking status					0.543
No	490 (84.19%)	480 (82.47%)	472 (81.10%)	486 (83.36%)	
Yes	92 (15.81%)	102 (17.53%)	110 (18.90%)	97 (16.64%)	
SBP (mmHg)	131.74 (23.33)	128.99 (24.99)	128.17 (24.47)	124.11 (24.91)	<0.001
DBP (mmHg)	76.81 (15.00)	76.03 (16.04)	76.86 (16.82)	75.06 (16.08)	0.183
LVEF (%)	49.00 (40.00-57.00)	47.00 (36.00-56.00)	45.00 (35.00-55.00)	44.00 (35.00-55.00)	<0.001
WBC (×10^9^/L)	5.80 (4.60-7.61)	6.10 (4.90-7.70)	6.30 (5.00-7.87)	6.70 (5.30-9.00)	<0.001
RBC (×10^12^/L)	3.99 (0.68)	4.08 (0.75)	4.12 (0.77)	4.09 (0.89)	0.033
PLT (×10^9^/L)	160.00 (124.00-205.00)	162.00 (126.00-208.00)	167.00 (130.00-219.00)	170.00 (128.00-221.50)	0.023
Alb (g/L)	37.00 (4.83)	35.75 (4.46)	35.32 (4.81)	33.82 (5.35)	<0.001
ALT (U/L)	20.00 (13.00-33.00)	20.00 (13.00-34.25)	21.00 (14.00-38.00)	25.00 (15.00-49.75)	<0.001
AST (U/L)	26.00 (20.00-36.00)	24.00 (19.00-35.00)	25.00 (19.00-38.00)	29.00 (20.00-48.00)	0.001
GGT (U/L)	38.00 (24.00-70.00)	40.00 (23.00-67.00)	42.00 (26.00-76.00)	46.00 (28.00-83.00)	<0.001
Cr (umol/L)	81.00 (65.00-107.00)	88.00 (69.00-121.00)	92.00 (75.00-127.50)	102.00 (77.00-145.75)	<0.001
BUN (mmol/L)	6.89 (5.44-9.26)	7.04 (5.45-9.78)	7.52 (5.78-10.35)	8.73 (6.31-13.66)	<0.001
UA (umol/L)	396.00 (314.00-490.00)	411.00 (327.00-507.00)	441.00 (353.25-555.00)	486.50 (373.25-612.75)	<0.001
TG (mmol/L)	0.92 (0.73-1.21)	1.09 (0.84-1.37)	1.24 (0.95-1.67)	1.41 (1.04-1.96)	<0.001
TC (mmol/L)	4.17 (3.56-4.80)	3.79 (3.26-4.41)	3.68 (3.16-4.38)	3.21 (2.64-3.98)	<0.001
HDL-C(mmol/L)	1.40 (0.21)	1.07 (0.09)	0.89 (0.08)	0.65 (0.13)	<0.001
LDL-C (mmol/L)	2.24 (1.76-2.79)	2.25 (1.76-2.81)	2.31 (1.84-2.96)	2.08 (1.57-2.65)	0.247
FPG (mmol/L)	5.10 (4.60-5.80)	5.30 (4.60-6.00)	5.40 (4.70-6.40)	5.60 (4.90-6.95)	<0.001
NT-proBNP (pmol/L)	3220.00 (1522.75-5247.00)	3480.00 (1877.75-6098.75)	3825.00 (1850.25-6705.25)	4219.00 (2023.00-7262.50)	<0.001

TyG/HDL-C ratio, triglyceride-glucose index/high-density lipoprotein cholesterol ratio; CHD, coronary heart disease; NYHA, New York Heart Association; LVEF: left ventricular ejection fraction; TG, triglyceride; TC, total cholesterol; HDL-C, high-density lipoprotein cholesterol; LDL-C, low-density lipid cholesterol; Cr, creatinine; BUN, Blood Urea Nitrogen; WBC, white blood cell count; RBC, red blood cell count; PLT, platelet count; ALT, alanine aminotransferase; AST, aspartate aminotransferase; GGT, Gamma-GlutamylTransferase; Alb, albumin; NT-proBNP, N-Terminal Pro-Brain Natriuretic Peptide; UA, uric acid; FPG, fasting plasma glucose.

### Follow-up results

**Figure 2:**
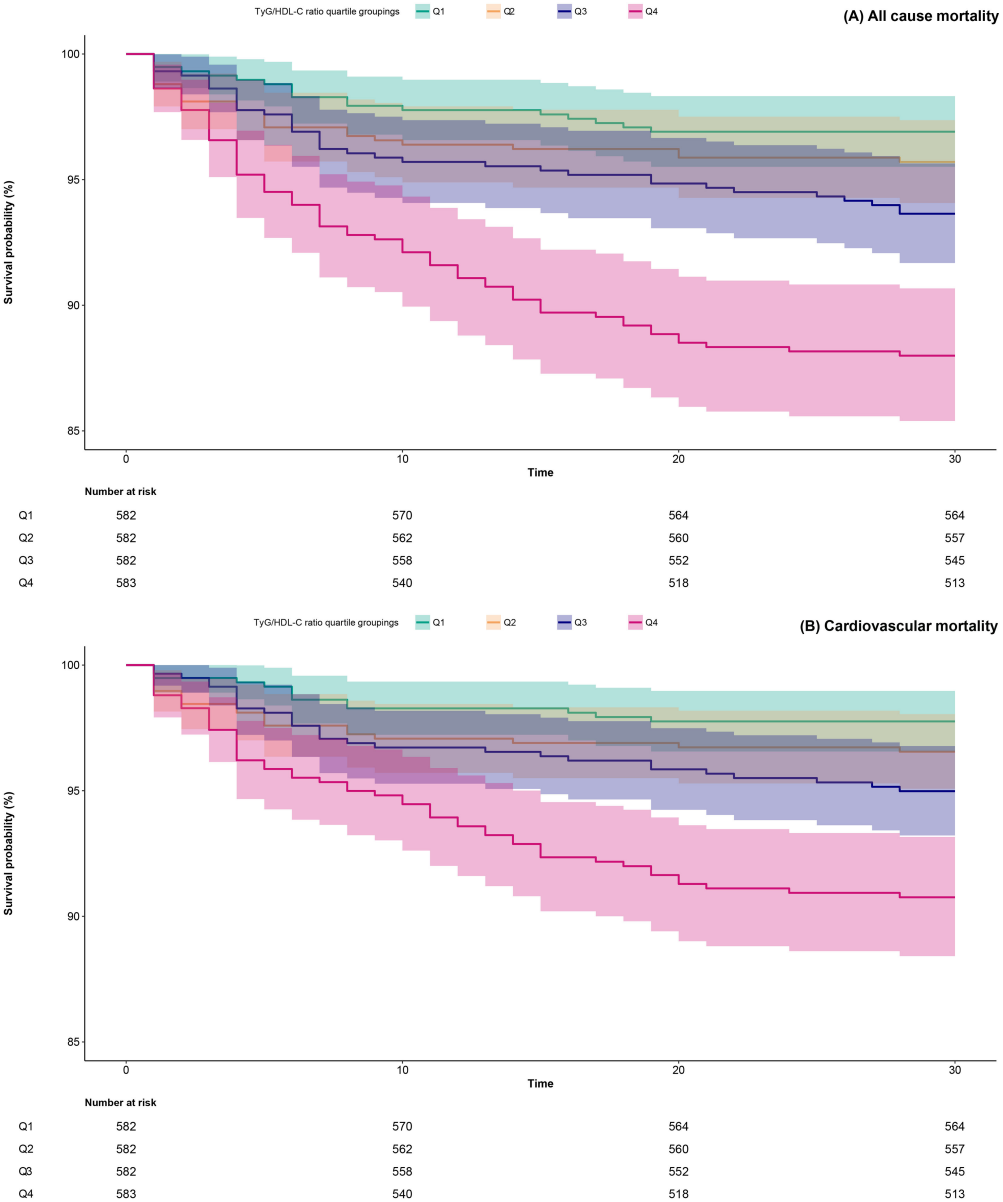
30-day survival curves of ADHF patients stratified by TyG/HDL-C ratio quartiles. TyG/HDL-C ratio: triglyceride-glucose index/high-density lipoprotein cholesterol ratio; ADHF: acute decompensated heart failure.

Within a 30-day follow-up of 2,329 ADHF patients, 6.44% (n=150) succumbed to mortality, including 115 cases linked to cardiovascular etiology. **Supplementary Figure S2** illustrates the stacked bar graph of TyG/HDL-C ratio quartiles and their association with all-cause mortality and cardiovascular mortality in ADHF patients. Notably, compared to patients in the low and intermediate RC groups, those in the high RC group showed significantly higher risks of both all-cause and cardiovascular mortality. Additionally, survival analysis stratified by TyG/HDL-C ratio quartiles revealed that the high RC group exhibited significantly higher risks of both all-cause mortality and cardiovascular mortality compared to the low and intermediate RC groups (**Figure 2**, Log-rank *p*<0.0001). Together, these results indicate that high RC may serve as an important risk factor for mortality in ADHF patients.

### The association between TyG/HDL-C ratio and 30-day mortality in patients with ADHF

The multivariable Cox regression analysis (**Table 2**) demonstrated that the TyG/HDL-C ratio was positively associated with 30-day mortality risk in patients with ADHF. In Cox regression models with stepwise adjustment for confounders (demographic characteristics → comorbidities and cardiac function → hematological factors), the effect size of the TyG/HDL-C ratio as a continuous variable gradually decreased but remained statistically significant. Final model (Model III) results indicated that for each 1-SD increment in TyG/HDL-C ratio, the all-cause mortality risk within 30 days for ADHF patients increased by 24% [HR: 1.24, 95% confidence interval (CI): 1.12, 1.36], while cardiovascular mortality risk increased by 20% (HR: 1.20, 95% CI: 1.08, 1.35). When analyzed by RC quartile groups, compared to Q1 group, Q4 group ADHF patients demonstrated a 218% increase in all-cause mortality risk within 30 days (HR: 3.18; 95% CI: 1.71, 5.93) and a 140% elevation in cardiovascular mortality risk (HR: 2.40, 95% CI: 1.11, 5.17), with an overall significant positive correlation trend (All *P*-trend<0.05).

**Table 2:** Multivariable Cox regression analysis of the association between TyG/HDL-C ratio and 30-day all-cause and cardiovascular mortality in patients with ADHF.

Independent variable	HR (95%CI)			
	Non-adjusted	Model I	Model II	Model III
All-cause mortality				
TyG/HDL-C ratio (Per SD increase)	1.42 (1.34, 1.52)	1.44 (1.35, 1.54)	1.33 (1.24, 1.44)	1.24 (1.12, 1.36)
TyG/HDL-C ratio (quartiles)				
Q1	Ref	Ref	Ref	Ref
Q2	1.40 (0.77, 2.57)	1.49 (0.81, 2.74)	1.39 (0.74, 2.60)	1.64 (0.86, 3.11)
Q3	2.08 (1.19, 3.66)	2.29 (1.30, 4.02)	2.04 (1.14, 3.64)	2.43 (1.31, 4.52)
Q4	4.05 (2.41, 6.80)	4.79 (2.84, 8.10)	3.41 (1.96, 5.92)	3.18 (1.71, 5.93)
*P*-trend	<0.0001	<0.0001	<0.0001	0.0001
Cardiovascular mortality				
TyG/HDL-C ratio (Per SD increase)	1.42 (1.32, 1.53)	1.43 (1.33, 1.54)	1.33 (1.22, 1.44)	1.20 (1.08, 1.35)
TyG/HDL-C ratio (quartiles)				
Q1	Ref	Ref	Ref	Ref
Q2	1.55 (0.77, 3.12)	1.60 (0.79, 3.22)	1.55 (0.75, 3.22)	1.66 (0.79, 3.51)
Q3	2.26 (1.17, 4.35)	2.38 (1.23, 4.59)	2.11 (1.07, 4.17)	2.15 (1.03, 4.52)
Q4	4.25 (2.32, 7.80)	4.68 (2.53, 8.65)	3.31 (1.72, 6.37)	2.40 (1.11, 5.17)
*P*-trend	<0.0001	<0.0001	<0.0001	0.0264

ADHF, acute decompensated heart failure; SD, standard deviation; TyG/HDL ratio, triglyceride glucose/high-density lipoprotein cholesterol ratio.

Model I adjusted for: Gender, age, drinking status, smoking status; Model II adjusted for: Gender, age, drinking status, smoking status, hypertension, diabetes, stroke, CHD, NYHA classification, and LVEF;

Model III adjusted for: Gender, age, drinking status, smoking status, hypertension, diabetes, stroke, CHD, NYHA classification, LVEF, WBC, RBC, PLT, Alb, AST, GGT, Cr, BUN, UA, TC, LDL-C, NT-proBNP.

We further examined the association between the TyG/HDL-C ratio and non-cardiovascular mortality in ADHF patients. As detailed in **Supplementary Table S5**, the TyG/HDL-C ratio demonstrated a borderline significant association with non-cardiovascular mortality in ADHF patients (HR: 1.26, 95% CI: 0.99–1.61; *P* = 0.0651).

### Dose-response relationship between TyG/HDL-C ratio and 30-day mortality in patients with ADHF

**Figure 3:**
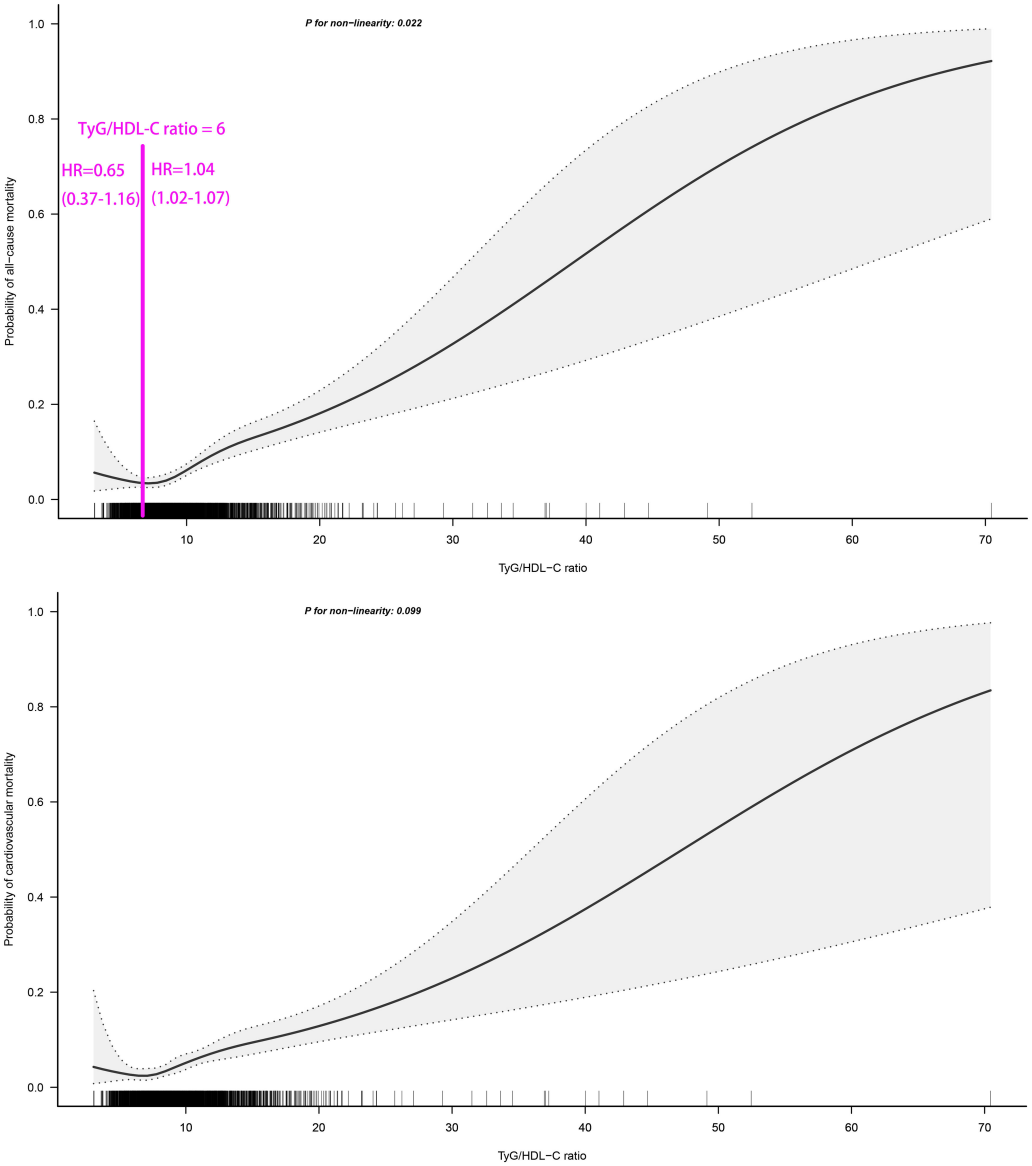
Fitting the dose-response relationship between TyG/HDL-C ratio and 30-day all-cause/cardiovascular mortality in ADHF patients with 4 knots restricted cubic spline. TyG/HDL-C ratio: triglyceride-glucose index/high-density lipoprotein cholesterol ratio; ADHF: acute decompensated heart failure. Adjusted for gender, age, drinking status, smoking status, hypertension, diabetes, stroke, CHD, LVEF, WBC, RBC, PLT, AST, GGT, Alb, Cr, BUN, UA, TC, LDL-C, NT-proBNP.

We employed RCS fitting and visualization to analyze the dose-response relationship between TyG/HDL-C ratio and 30-day mortality in ADHF patients. As illustrated in **Figure 3**, the TyG/HDL-C ratio showed a non-linear positive correlation with 30-day all-cause mortality risk in ADHF patients (*P* for non-linearity=0.022), while demonstrating a borderline non-linear association with cardiovascular mortality risk (*P* for non-linearity=0.099). Through two-piecewise Cox regression, we observed that all-cause mortality risk was inversely correlated with TyG/HDL-C ratio before reaching the threshold of 6, after which a positive correlation emerged.

### Exploratory analysis of the combined effects of TyG index and HDL-C on 30-day mortality risk in patients with ADHF

**Figure 4:**
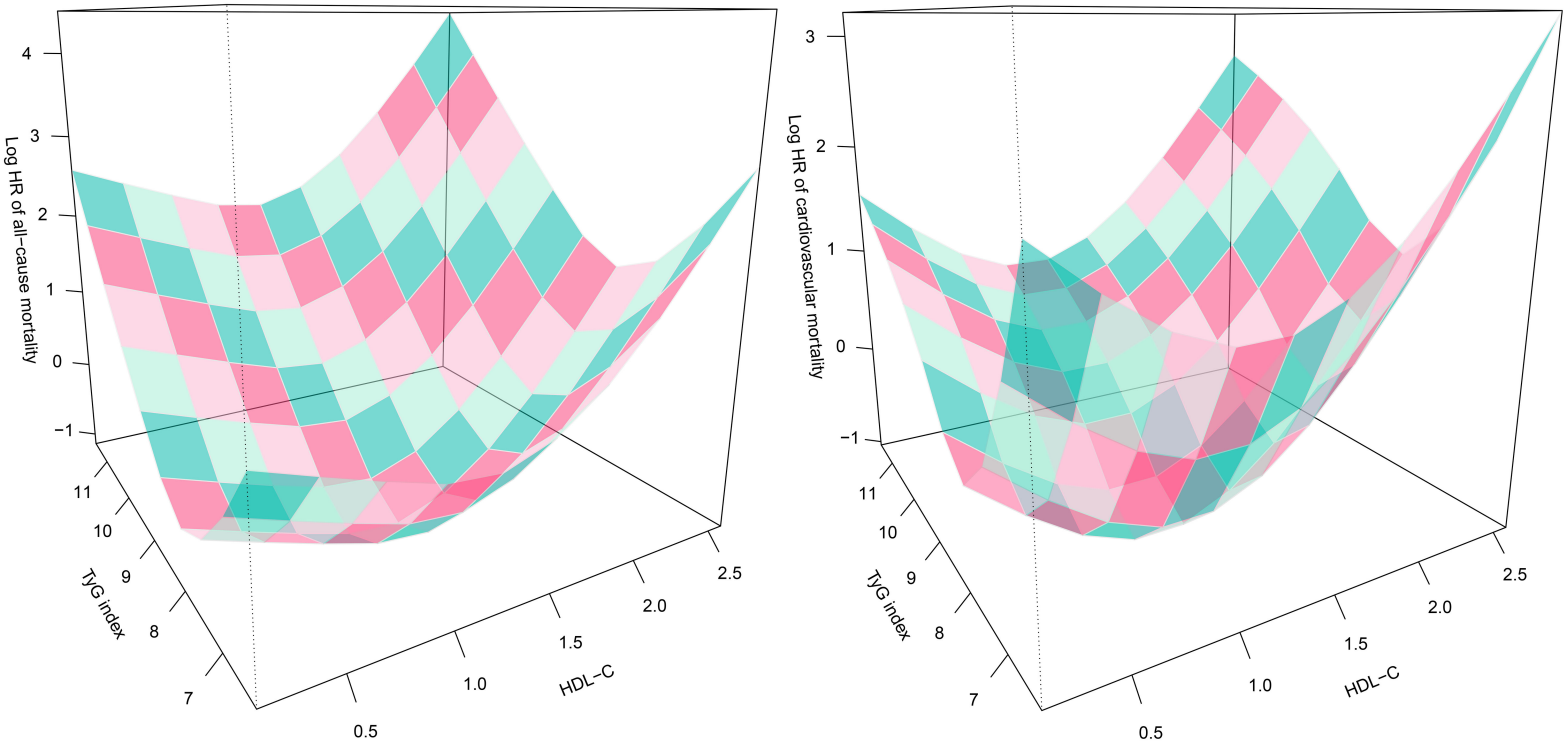
Three-dimensional surface plots of TyG index, HDL-C levels, and 30-day all-cause/cardiovascular mortality in ADHF patients. TyG/HDL-C ratio: triglyceride-glucose index/high-density lipoprotein cholesterol ratio; ADHF: acute decompensated heart failure. Adjusted for gender, age, drinking status, smoking status, hypertension, diabetes, stroke, CHD, LVEF, WBC, RBC, PLT, AST, GGT, Alb, Cr, BUN, UA, TC, LDL-C, NT-proBNP.

Based on the adjustment strategy of Model III, we further evaluated the combined effects of TyG index and HDL-C with 30-day mortality in ADHF patients. Utilizing OpenGL technology, we generated a 3D surface plot (**Figure 4**) visualizing the relationship between TyG index, HDL-C, and 30-day mortality. The study revealed a unique dose-response relationship in their combined effect—a complex U-shaped pattern resembling the concave surface of a radio telescope. Overall, under the combined effect, both low and high combinations of TyG index and HDL-C indicated increased risks of 30-day all-cause mortality and cardiovascular mortality in ADHF patients, suggesting the bidirectional risk characteristics of metabolic homeostasis imbalance. Concurrently, when the TyG index and HDL-C levels are maintained within specific ranges, the 30-day mortality risk in ADHF patients is minimized. Preliminary estimates indicate that when the TyG index is maintained between 7.5 and 9.0, and HDL-C levels between 1.0 and 1.5 mmol/L, the 30-day mortality risk in ADHF patients is relatively low.

### Subgroup analysis

**Table 3** presents the analysis results stratified by age, gender, LVEF, NYHA classification, and comorbidities. After further comparisons between strata using the likelihood ratio test, the study identified LVEF as a potentially important modifier in the association between TyG/HDL-C ratio and all-cause mortality in ADHF patients. There was a borderline interaction between LVEF and all-cause mortality related to TyG/HDL-C ratio (*P* for interaction = 0.0873). Specifically, compared to patients with LVEF ≥50%, the effect of TyG/HDL-C ratio on all-cause mortality risk was relatively higher in patients with LVEF <50% (1.42 vs. 1.18).

**Table 3:** Stratified analysis showed the relationship between TyG/HDL-C ratio and 30-day mortality in patients with ADHF in different age, gender, NYHA class, LVEF and whether combined with hypertension/diabetes/cerebral infarction/CHD.

Subgroup	HR Per SD increase (95%CI)	
	All-cause mortality	Cardiovascular mortality
Age (years)		
19-68	1.38 (1.17, 1.62)	1.33 (1.09, 1.62)
69-99	1.19 (1.06, 1.33)	1.17 (1.02, 1.32)
*P* for interaction	0.1451	0.2841
Gender		
Male	1.27 (1.15, 1.41)	1.24 (1.11, 1.40)
Female	1.13 (0.94, 1.35)	1.07 (0.86, 1.33)
*P* for interaction	0.2156	0.1854
NYHA		
III	1.13 (0.92, 1.40)	1.22 (0.97, 1.54)
IV	1.26 (1.14, 1.38)	1.20 (1.06, 1.35)
*P* for interaction	0.3364	0.8734
LVEF		
< 50%	1.42 (1.19, 1.68)	1.30 (1.06, 1.60)
≥ 50%	1.18 (1.06, 1.32)	1.18 (1.03, 1.34)
*P* for interaction	0.0873	0.4079
Hypertension		
Yes	1.33 (1.11, 1.58)	1.18 (0.89, 1.55)
No	1.21 (1.09, 1.35)	1.21 (1.07, 1.36)
*P* for interaction	0.3858	0.8547
Diabetes		
Yes	1.32 (1.07, 1.64)	1.15 (0.83, 1.58)
No	1.22 (1.10, 1.36)	1.21 (1.08, 1.35)
*P* for interaction	0.5033	0.7454
Stroke		
Yes	1.13 (0.94, 1.37)	1.10 (0.86, 1.42)
No	1.26 (1.15, 1.40)	1.22 (1.09, 1.37)
*P* for interaction	0.2685	0.4248
CHD		
Yes	1.23 (1.09, 1.38)	1.19 (1.04, 1.37)
No	1.26 (1.09, 1.45)	1.22 (1.03, 1.43)
*P* for interaction	0.7720	0.8406

TyG/HDL-C ratio, triglyceride-glucose index/high-density lipoprotein cholesterol ratio; ADHF, acute decompensated heart failure; CHD, coronary heart disease.

Models adjusted for the same covariates as in model III (**Table 2**), except for the stratification variable.

### Comparative predictive value of TyG/HDL-C ratio vs. TyG index for 30-day mortality in ADHF patients

Receiver operating characteristic curve analysis confirmed (**Table 4**) that the TyG/HDL-C ratio demonstrated superior predictive performance compared to the TyG index in predicting 30-day mortality in ADHF patients. Specifically, compared to the TyG index, the TyG/HDL-C ratio showed higher AUC values in predicting both 30-day all-cause and cardiovascular mortality risks in ADHF patients (**Supplementary Figure S3**), with the best threshold of 9.78 for both.

**Table 4:** ROC analysis compares the predictive value of the TyG index and the TyG/HDL-C ratio for 30-day all-cause mortality and cardiovascular mortality.

Variable	AUC	95%CI low	95%CI upp	Best threshold	Specificity	Sensitivity	C-index
All-cause mortality							
TyG index*	0.62	0.57	0.67	8.91	0.77	0.46	0.61
TyG/HDL-C ratio	0.67	0.62	0.71	9.78	0.65	0.61	0.66
Cardiovascular mortality							
TyG index*	0.59	0.53	0.64	8.94	0.77	0.41	0.59
TyG/HDL-C ratio	0.66	0.61	0.72	9.78	0.64	0.62	0.66

AUC, area under the curve; other abbreviations as in **Table 1**. *P<0.05, compare with TyG/HDL-C ratio.

Additionally, we calculated the C-index to further validate the predictive consistency of the TyG/HDL-C ratio for mortality risk in ADHF patients, and our analyses revealed that the AUC and C-index demonstrated consistent and complementary results, collectively supporting the robustness of the TyG/HDL-C ratio for mortality prediction in ADHF patients (**Table 4**).

### Incremental predictive performance of adding the TyG/HDL-C ratio to the NT-proBNP model for mortality risk assessment

When adding the TyG/HDL-C ratio to the NT-proBNP-based model for predicting 30-day mortality, we observed a significant improvement in the model’s predictive performance for mortality outcomes (**Supplementary Table S6**). For all-cause mortality prediction, the C-index increased from 0.67 to 0.72 (*P* < 0.01), with an NRI of 0.19 (*P* = 0.01). For cardiovascular mortality prediction, the C-index increased from 0.71 to 0.75 (*P* < 0.01), with an NRI of 0.15 (*P* = 0.04). These findings suggest that incorporating the TyG/HDL-C ratio into the NT-proBNP model confers a significant incremental benefit for short-term mortality risk prediction.

### Exploratory mediation analysis

**Figure 5:**
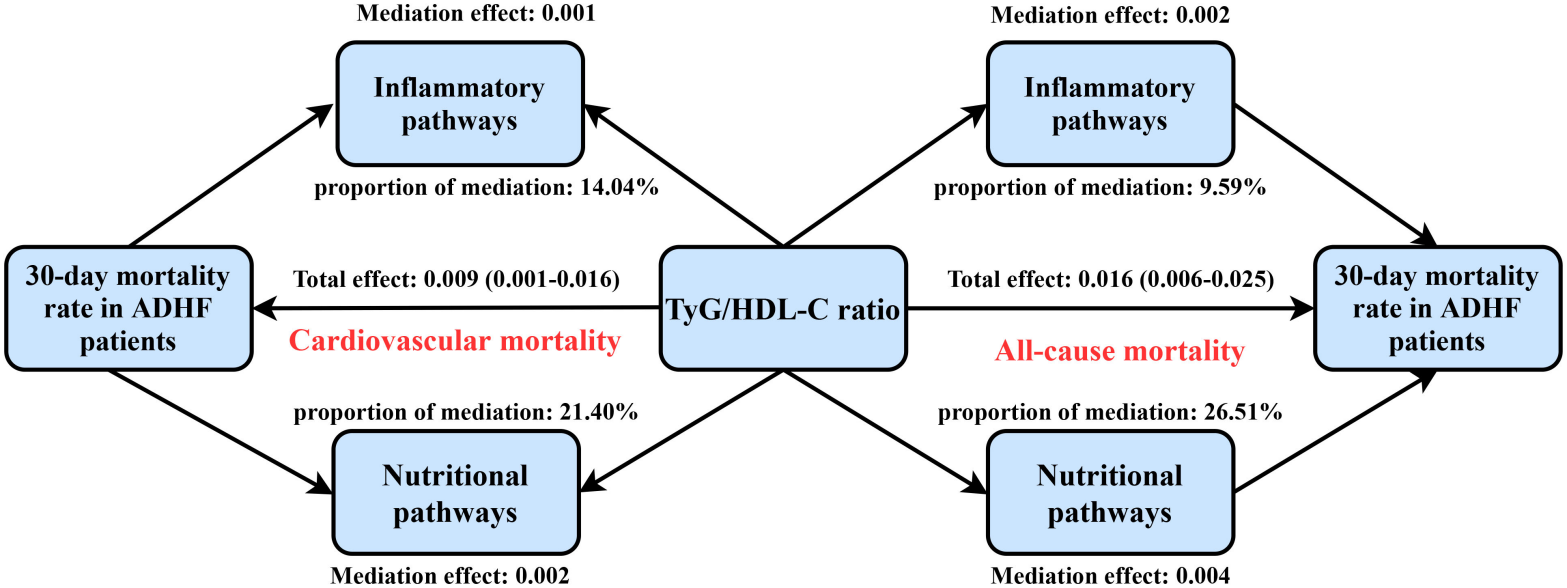
Path diagram for mediational model. TyG/HDL-C ratio: triglyceride-glucose index/high-density lipoprotein cholesterol ratio; ADHF: acute decompensated heart failure. Adjusted for gender, age, drinking status, smoking status, hypertension, diabetes, stroke, CHD, LVEF, RBC, PLT, AST, Cr, BUN, UA, TC, LDL-C, NT-proBNP.

Mediation analysis based on the Bootstrap method revealed substantial pathway heterogeneity in the association between TyG/HDL-C ratio and 30-day mortality risk in ADHF patients (**Supplementary Table S7**). The study demonstrated (**Figure 5**) that the inflammatory pathway mediated 9.59% of the all-cause mortality risk (*P*-value of proportion mediate = 0.004) and 14.04% of the cardiovascular mortality risk (*P*-value of proportion mediate = 0.024), while the nutritional metabolic pathway contributed to 26.51% of the all-cause mortality risk (*P*-value of proportion mediate < 0.001). Notably, the oxidative stress pathway showed no statistically significant mediation effect for either mortality endpoint.

### Sensitivity analysis

Sensitivity analysis confirmed that the association between TyG/HDL-C ratio and 30-day all-cause and cardiovascular mortality risks in ADHF patients remained robust under different methodological assumptions (**Table 5**). Sensitivity-1: After adding SBP, DBP, TG, and FPG (which exhibited no multicollinearity) to the final model, the TyG/HDL-C ratio remained stably associated with 30-day mortality in ADHF patients. Sensitivity-2: After excluding cases of death within 48 hours after admission to avoid reverse causality, the corresponding risk gradients also remained stable. Sensitivity-3: Additionally, to exclude the impact of frailty on mortality risk, we conducted a sensitivity analysis after excluding the frail subgroup, and the results remained consistent. Sensitivity-4: To mitigate the potential impact of missing data on the results, we employed the multiple imputation method to handle missing data and repeated the core analysis, yielding similar results. These validation analyses systematically excluded the influence of potential biases on the conclusions from four dimensions: model construction, causal timing, population heterogeneity, and data integrity. Sensitivity-5: After further adjusting for inpatient HF medications, the results remained consistent with the primary findings. Sensitivity-6 and 7: When defining the study outcome as 20-day and 25-day mortality, the results did not show substantial fluctuations compared to the 30-day mortality analysis. Sensitivity-8: The heatmap illustrating the association between the TyG index, HDL-C, and 30-day mortality in ADHF patients (**Supplementary Figure S4**) revealed findings consistent with the 3D surface plot: ADHF patients exhibited a relatively lower risk of 30-day mortality when TyG index levels were maintained between 7.5–9.0 and HDL-C levels between 1.0–1.5 mmol/L.

**Table 5:** Sensitivity analysis.

Independent variable	Hazard ratios (95% confidence interval)						
	Sensitivity-1	Sensitivity-2	Sensitivity-3	Sensitivity-4	Sensitivity-5	Sensitivity-6	Sensitivity-7
All-cause mortality							
TyG/HDL-C ratio (Per SD increase)	1.20 (1.09, 1.33)	1.22 (1.09, 1.36)	1.23 (1.12, 1.36)	1.27 (1.16, 1.38)	1.16 (1.06, 1.27)	1.24 (1.13, 1.36)	1.24 (1.13, 1.36)
TyG/HDL-C ratio (quartiles)							
Q1	Ref	Ref	Ref	Ref	Ref	Ref	Ref
Q2	1.49 (0.78, 2.85)	1.27 (0.62, 2.60)	1.52 (0.78, 2.95)	1.69 (0.91, 3.16)	2.43 (1.25, 4.75)	1.62 (0,85, 3.10)	1.60 (0.84, 3.07)
Q3	2.15 (1.15, 4.01)	2.21 (1.13, 4.33)	2.30 (1.22, 4.32)	2.45 (1.34, 4.50)	2.72 (1.46, 5.07)	2.02 (1.06, 3.83)	2.18 (1.16, 4.10)
Q4	2.81 (1.49, 5.29)	2.95 (1.49, 5.84)	2.97 (1.57, 5.63)	3.61 (1.98, 6.58)	3.27 (1.76, 6.09)	3.20 (1.71, 5.99)	3.20 (1.71, 6.00)
*P*-trend	0.0006	0.0004	0.0004	<0.0001	0.0003	0.0002	0.0001
Cardiovascular mortality							
TyG/HDL-C ratio (Per SD increase)	1.19 (1.06, 1.33)	1.22 (1.07, 1.40)	1.20 (1.08, 1.35)	1.24 (1.12, 1.37)	1.13 (1.01, 1.27)	1.20 (1.08, 1.34)	1.20 (1.07, 1.34)
TyG/HDL-C ratio (quartiles)							
Q1	Ref	Ref	Ref	Ref	Ref	Ref	Ref
Q2	1.52 (0.72, 3.21)	1.44 (0.62, 3.36)	1.56 (0.72, 3.34)	1.60 (0.78, 3.29)	2.34 (1.08, 5.07)	1.58 (0.74, 3.36)	1.57 (0.73, 3.34)
Q3	1.89 (0.89, 4.00)	2.65 (1.20, 5.87)	1.88 (0.87, 4.02)	2.09 (1.02, 4.27)	2.31 (1.10, 4.87)	1.76 (0.82, 3.78)	1.95 (0.92, 4.13)
Q4	2.15 (0.98, 4.68)	3.04 (1.34, 6.89)	2.19 (1.00, 4.83)	2.63 (1.27, 5.45)	2.70 (1.24, 5.87)	2.25 (1.03, 4.91)	2.28 (1.05, 4.96)
*P*-trend	0.0358	0.0029	0.0437	0.0072	0.0299	0.0459	0.0373

ADHF, acute decompensated heart failure; SD, standard deviation; TyG/HDL ratio, triglyceride glucose/high-density lipoprotein cholesterol ratio.

Adjusted Gender, age, drinking status, smoking status, hypertension, diabetes, stroke, CHD, NYHA classification, LVEF, WBC, RBC, PLT, Alb, AST, GGT, Cr, BUN, UA, TC, LDL, NT-proBNP.

Hypertension, diabetes, stroke and CHD were not adjusted in Sensitivity-3.

Sensitivity-5 adjusted gender, age, drinking status, smoking status, hypertension, diabetes, stroke, CHD, NYHA classification, LVEF, WBC, RBC, PLT, Alb, AST, GGT, Cr, BUN, UA, TC, LDL, NT-proBNP, beta-blockers, diuretics, ACEI/ ARB/ARNI, digitalis, SGTL-2, and vasopressor medications.

## Discussion

This study confirmed a significant positive correlation between elevated TyG/HDL-C ratio and 30-day all-cause and cardiovascular mortality risks in ADHF patients in the Jiangxi, China ADHF patient cohort, and subsequently evaluated the robustness of the research findings through multidimensional sensitivity analysis; these discoveries filled an evidence gap for this indicator in region-specific populations. More importantly, through 3D interaction model analysis, we unveiled the complexity of the interaction patterns between TyG index and HDL-C in shaping 30-day mortality prognosis for ADHF patients. This bidirectional risk profile suggests limitations of conventional target management strategies based on linear thresholds. Clinically, it is imperative to implement dynamic monitoring of the synchronized fluctuation window between TyG index and HDL-C levels to achieve precision modulation of metabolic homeostasis, thereby providing personalized intervention pathways for improving short-term prognosis in ADHF patients.

As the critical stage of HF, ADHF carries an in-hospital mortality rate of approximately 10%, posing a central challenge in global cardiovascular disease management [4.5]. Recent studies have demonstrated that glucolipid metabolic disorders profoundly contribute to ADHF pathological progression through mechanisms such as IR and lipoprotein dysfunction, thereby significantly impacting disease prognosis (10–12, 21, 22). The TyG index serves as a reliable surrogate marker for IR, with multicenter cohort studies validating its strong correlation with prognosis across various HF subtypes. In mortality risk assessment, Han et al. found that each 1-unit increase in TyG index conferred an 88.6% elevated all-cause in-hospital mortality risk among HF patients (17). The research team led by Zhou et al. revealed through longitudinal studies on Chinese chronic HF populations that patients in the highest TyG index tertile demonstrated 84% higher all-cause mortality risk and 94% higher cardiovascular mortality risk compared with the lowest tertile (16). Furthermore, across different HF subtypes and specific therapeutic populations, the TyG index consistently maintained comparable risk association patterns with mortality prognosis in HF patients. Notably, this association has been corroborated across diverse ethnic groups (14, 15, 18–20). From the lipid metabolism perspective, functional assessment of HDL-C demonstrates superior clinical value compared to traditional concentration-based metrics. A nested case-control study revealed that each 1-SD increase in small-particle HDL-C concentration conferred a 65% lower risk of 3-month all-cause mortality among acute HF patients (34). Further functional analyses confirmed that every 10% enhancement in HDL-C-mediated cholesterol efflux capacity was accompanied by a 22% reduction in in-hospital mortality risk, underscoring the pivotal role of HDL functional restoration in metabolic interventions (35). These evidences collectively indicate that metabolic dysregulation biomarkers (TyG index and HDL-C) not only deepen our understanding of ADHF pathogenesis, but also introduce novel insights for personalized treatment through quantitative risk stratification.

Glucolipid metabolic disorders, particularly abnormalities in the TyG index and HDL-C levels, are significant contributors to adverse prognosis in ADHF patients (14–20, 30, 31). Building on this evidence, our study innovatively integrates the TyG index with HDL-C to establish the TyG/HDL-C ratio. Our latest findings reveal a significant positive association between the TyG/HDL-C ratio and both all-cause mortality and cardiovascular mortality risks in ADHF. Notably, despite prior studies establishing NT-proBNP (36–38) and hepatorenal biomarkers (39–41) as core indicators for ADHF risk assessment, the TyG/HDL-C ratio maintained its independent predictive value even after adjusting for confounders including NT-proBNP, renal function (Cr, BUN), and hepatic function (AST, GGT, Alb). As a novel metabolic syndrome-derived index, previous studies on the TyG/HDL-C ratio have mainly focused on metabolic diseases. For instance, Tong et al. identified an independent association between elevated TyG/HDL-C ratio and coronary artery calcification risk, exhibiting notably enhanced predictive accuracy for CAC in the elderly subgroup (≥60 years) compared to conventional metrics (24). Similarly, Gao et al. revealed that in a diabetes population, an increased TyG/HDL-C ratio was linked to a 3.16-fold elevated risk of metabolic dysfunction-associated fatty liver disease (23). These evidences not only corroborate the reliability of the TyG/HDL-C ratio in assessing metabolic dysregulation, but also provides a preliminary theoretical foundation for extending this metric to ADHF prognostic prediction in our study.

In the current study, our mediation analysis further revealed that nutritional and inflammatory pathways may play significant mediating roles in TyG/HDL-C ratio-associated mortality risk in ADHF patients. Data indicate that the inflammatory pathway mediated approximately 9.59% and 14.04% of the TyG/HDL-C ratio-associated risks for all-cause mortality and cardiovascular mortality, respectively. In contrast, the nutritional pathway exerted a more substantial influence, mediating approximately 26.51% of the TyG/HDL-C ratio-associated risk for all-cause mortality. This finding indicates the important role of nutritional and inflammatory pathways in mortality risk assessment (42–46). Targeted interventions to reduce indirect contributing factors (e.g., nutritional support and anti-inflammatory therapies) may improve clinical outcomes in ADHF patients with elevated TyG/HDL-C ratios (42, 47, 48). For nutritional supplementation in ADHF patients, while existing literature provides varying levels of evidence supporting different approaches, individualized treatment remains a critical factor; it is recommended that dietitians be involved early in the management of ADHF patients with elevated TyG/HDL-C ratios upon admission (49, 50). Regarding anti-inflammatory therapies for ADHF, current evidence supports potential benefits of enhanced anti-inflammatory treatment for the majority of patients (51–53); however, further research evidence is still required to validate widely applicable anti-inflammatory interventions (54). While the specific mechanisms underlying the association between TyG/HDL-C ratio and adverse ADHF outcomes remain unclear, we propose, based on the biological properties of its components and mediation analysis findings, that this association may be linked to secondary inflammatory responses, energy metabolic dysregulation, and subsequent nutritional deterioration when TyG index and HDL-C act synergistically (10–12, 22).

In the final model with full adjustment for confounders, our study, through RCS and 3D interaction model analysis, reveals for the first time the unique dose-dependent association patterns between the TyG/HDL-C ratio (and its components) and both all-cause and cardiovascular mortality risks in ADHF. The findings indicate a nonlinear positive correlation between TyG/HDL-C ratio and 30-day all-cause mortality risk in ADHF patients, as well as a borderline nonlinear association with cardiovascular mortality risk. Additionally, the 3D interaction model analysis uncovered a complex U-shaped-concave association in the combined effect of TyG index and HDL-C: both excessively low and high combinations of TyG index and HDL-C were linked to increased 30-day all-cause and cardiovascular mortality risks in ADHF patients. Concurrently, when TyG index (7.5-9.0) and HDL-C (1.0-1.5 mmol/L) were kept within specific ranges, the 30-day mortality risk in ADHF patients was minimized. These discoveries challenge the assumptions of traditional linear risk models and provide novel evidence for metabolic management in ADHF. Previous studies have repeatedly demonstrated a “U-shaped” association between TyG index levels and various chronic diseases as well as their adverse prognosis risks in diverse patient populations (55–64), with the inflection point approximately located around 9, consistent with our study findings. Evidence suggests that excessively low TyG index levels are significantly associated with adverse health outcomes, and the mechanisms may involve multidimensional pathophysiological pathways, including the activation of the sympathetic-adrenal axis and metabolic imbalances (65, 66). Moreover, the severe IR state reflected by high TyG index further amplifies mortality risks through chronic inflammation, oxidative stress, and disrupted energy metabolism (10–12). This nonlinear association reveals that both hyperactivation and dysfunction of insulin signaling pathways can disrupt metabolic homeostasis, suggesting that clinical interventions should be based on individualized thresholds (e.g., TyG index inflection point ≈9.0) rather than solely targeting the “normalization” of biochemical indicators. Traditionally, HDL-C has been widely recognized as the body’s “good cholesterol” (67), effectively reducing the accumulation of excess cholesterol through reverse cholesterol transport and preventing the formation of atherosclerosis (68, 69). However, recent large-scale cohort studies have overturned the conventional belief that “the higher the HDL-C concentration, the better,” revealing a U-shaped or J-shaped association between HDL-C levels and both all-cause and cardiovascular mortality risks. For instance, a recent study found that in patients with type 2 diabetes, both low HDL-C (<40 mg/dL) and high HDL-C (≥80 mg/dL) significantly increased the risk of major adverse cardiovascular events (HRs of 1.24 and 1.09, respectively), with the risk curve exhibiting a U-shaped distribution (*P* for non-linearity <0.001) (70). Furthermore, HDL-C-related nonlinear findings have been reported across diverse ethnic populations: a Japanese cohort revealed a 137% surge in atherosclerotic cardiovascular mortality risk when HDL-C ≥90 mg/dL (71); in the general British population, HDL-C >100 mg/dL was associated with 11%-24% increased risks of all-cause and cardiovascular mortality (72); a meta-analysis incorporating 17 studies further confirmed that high HDL-C (>80 mg/dL) uniformly increased all-cause mortality, cardiovascular mortality, and stroke risk by 14%-15% (73). Potential mechanisms may involve genetic variations such as SCARB1 carriage in individuals with extremely high HDL-C levels, as well as the HDL particle retention hypothesis (74, 75). These findings suggest that clinical practice should re-evaluate the “higher-is-better” monitoring strategy for HDL-C and explore functional HDL assessment systems to achieve precision risk stratification.

Subgroup analysis revealed a borderline significant positive difference in the association between the TyG/HDL-C ratio and all-cause mortality risk in ADHF patients across LVEF subgroups. Specifically, the TyG/HDL-C ratio showed a stronger association with all-cause mortality in ADHF patients with LVEF <50% compared to those with LVEF ≥50% (1.42 vs. 1.18, *P* for interaction = 0.0873). This finding echoes the U-shaped association between LVEF and mortality risk: numerous previous cohort studies have confirmed that when LVEF is between 50%-69.9%, there exists a significant bidirectional plateau in mortality improvement and deterioration, with both extremely high and low LVEF values significantly increasing risks (76–78). Notably, patients with LVEF <50% may further amplify the risk effects of the TyG/HDL-C ratio through dual pathological pathways: on one hand, skeletal muscle atrophy and mitochondrial oxidative phosphorylation dysfunction lead to peripheral IR deterioration, driving abnormal elevation of the TyG index (79–81); on the other hand, systemic inflammatory states and overactivation of the renin-angiotensin system contribute to depletion in both HDL-C levels and its anti-inflammatory function (82–84). Our study data further support this phenomenon, demonstrating that HDL-C levels were significantly lower in patients with LVEF <50% compared to those with LVEF ≥50% (0.98 ± 0.30 vs. 1.03 ± 0.31 mmol/L, *P*=0.012). The synergistic effects of these mechanisms may contribute to the relatively higher all-cause mortality risk observed in the LVEF <50% population.

The findings of this study offer potential novel strategies for the clinical management of ADHF. As a clinically common critical illness, ADHF is characterized by severe symptoms, acute onset, and poor short-term prognosis, making it one of the most challenging conditions in inpatient management (3–5). This study provides the first validation of the independent association between the novel metabolic biomarker TyG/HDL-C ratio and 30-day mortality risk in ADHF patients, confirming its superiority in short-term prognostic assessment: The TyG/HDL-C ratio not only significantly outperforms the traditional TyG index but also provides statistically significant incremental prognostic value beyond the NT-proBNP model. Notably, measurement of the TyG/HDL-C ratio offers practical advantages, including simplicity, low cost, and compatibility with existing laboratory tests in primary care settings. Based on these findings, we recommend incorporating the TyG/HDL-C ratio into risk-stratification systems for ADHF patients as a practical tool to optimize prognostic evaluation.

### Strengths and limitations

Strengths of this study include being the first to report the independent association between the TyG/HDL-C ratio and 30-day all-cause and cardiovascular mortality risks in a Chinese ADHF population. Notably, sensitivity analyses across four dimensions—model adjustment, causal timing, population heterogeneity, and data integrity—all demonstrated the high robustness of these findings. These discoveries address a critical regional evidence gap in short-term prognostic evaluation for ADHF. Additionally, through a 3D interaction model, we further elucidated the optimal metabolic homeostasis window for TyG and HDL-C in assessing mortality risk among ADHF patients. The threshold range associated with concave-shaped associations provides critical data for clinical development of personalized metabolic intervention strategies. Finally, the mediation analysis model quantified the mediating effects of inflammation and nutritional metabolism in the relationship between TyG/HDL-C and mortality risk, providing a theoretical basis for developing multi-dimensional therapeutic strategies targeting the inflammation-nutrition axis.

This study has several limitations: Firstly, as a regional cohort study, the generalizability of our findings to other racial or geographic populations warrants caution. Secondly, although we have included relevant confounding factors as much as possible, residual confounding from unmeasured variables may persist. Thirdly, the lack of systematic documentation on how pharmacological interventions regulate metabolic indices (TyG index and HDL-C) and prognosis may lead to an underestimation of the true effect size linking metabolism to mortality. Fourthly, the U-shaped association between the TyG/HDL-C ratio and mortality involves complex metabolic interactions. This finding warrants further validation in external cohorts, while the underlying biological mechanisms require in-depth exploration in future review studies. Fifthly, the 30-day follow-up window, while appropriate for evaluating acute-phase outcomes, provides limited insight into TyG/HDL-C ratio’s longitudinal prognostic implications for ADHF patients. Comprehensive investigations with prolonged observation periods are warranted to elucidate the temporal progression of TyG/HDL-C ratio’s effects across all clinical timelines. Finally, while the 30-day follow-up period effectively captures acute-phase mortality risk in ADHF, it may miss time-dependent associations of metabolic disorders with mid-to-long-term outcomes. Future studies with extended follow-up durations are planned to further analyze their dose-response relationship.

## Conclusion

In this cohort analysis, we validated that the TyG/HDL-C ratio serves as an independent predictor of 30-day all-cause and cardiovascular mortality risks in ADHF patients. Notably, through 3D interaction analysis, we identified a concave-shaped relationship between the combined effects of TyG index and HDL-C levels on mortality risk. This discovery establishes the first evidence-based threshold framework for precision metabolic management in ADHF. Building on these findings, we propose that formulating intervention strategies based on individualized thresholds, rather than rigidly pursuing “normalization” of biochemical markers, may better reduce short-term mortality risk in ADHF patients.

## Data Availability

The raw data supporting the conclusions of this article will be made available by the authors, without undue reservation.

## References

[1] Bozkurt B Coats AJS Tsutsui H Abdelhamid CM Adamopoulos S Albert N. Universal definition and classification of heart failure: a report of the Heart Failure Society of America, Heart Failure Association of the European Society of Cardiology, Japanese Heart Failure Society and Writing Committee of the Universal Definition of Heart Failure: Endorsed by the Canadian Heart Failure Society, Heart Failure Association of India, Cardiac Society of Australia and New Zealand, and Chinese Heart Failure Association. Eur J Heart Fail. (2021) 23:352–80. doi: 10.1002/ejhf.2115, PMID: 33605000

[2] Metra M Tomasoni D Adamo M Bayes-Genis A Filippatos G Abdelhamid M. Worsening of chronic heart failure: definition, epidemiology, management and prevention. A clinical consensus statement by the Heart Failure Association of the European Society of Cardiology. Eur J Heart Fail. (2023) 25:776–91. doi: 10.1002/ejhf.2874, PMID: 37208936

[3] Gheorghiade M Zannad F Sopko G Klein L Piña IL Konstam MA. Acute heart failure syndromes: current state and framework for future research. Circulation. (2005) 112:3958–68. doi: 10.1161/CIRCULATIONAHA.105.590091, PMID: 16365214

[4] Krumholz HM Merrill AR Schone EM Schreiner GC Chen J Bradley EH. Patterns of hospital performance in acute myocardial infarction and heart failure 30-day mortality and readmission. Circ Cardiovasc Qual Outcomes. (2009) 2:407–13. doi: 10.1161/CIRCOUTCOMES.109.883256, PMID: 20031870

[5] Bernheim SM Grady JN Lin Z Wang Y Wang Y Savage SV. National patterns of risk-standardized mortality and readmission for acute myocardial infarction and heart failure. Update on publicly reported outcomes measures based on the 2010 release. Circ Cardiovasc Qual Outcomes. (2010) 3:459–67. doi: 10.1161/CIRCOUTCOMES.110.957613, PMID: 20736442 PMC3027304

[6] Tay WT Teng TK Ouwerkerk W Angermann CE Dickstein K Cleland JGF. Quality of care delivery in patients with acute heart failure: insights from the international REPORT-HF registry. EClinicalMedicine. (2025) 80:103031. doi: 10.1016/j.eclinm.2024.103031, PMID: 39877260 PMC11773266

[7] Wang H Chai K Du M Wang S Cai JP Li Y. Prevalence and incidence of heart failure among urban patients in China: A national population-based analysis. Circ Heart Fail. (2021) 14:e008406. doi: 10.1161/CIRCHEARTFAILURE.121.008406, PMID: 34455858

[8] Zhang Y Gao C Greene SJ Greenberg BH Butler J Yu J. Clinical performance and quality measures for heart failure management in China: the China-Heart Failure registry study. ESC Heart Fail. (2023) 10:342–52. doi: 10.1002/ehf2.14184, PMID: 36224725 PMC9871659

[9] Xiang H Tao X Guan X Yin T Li J Dong D. Contemporary Chinese dietary pattern: Where are the hidden risks? Front Nutr. (2022) 9:997773. doi: 10.3389/fnut.2022.997773, PMID: 36211490 PMC9544811

[10] Lopaschuk GD Karwi QG Tian R Wende AR Abel ED. Cardiac energy metabolism in heart failure. Circ Res. (2021) 128:1487–513. doi: 10.1161/CIRCRESAHA.121.318241, PMID: 33983836 PMC8136750

[11] Riehle C Abel ED. Insulin signaling and heart failure. Circ Res. (2016) 118:1151–69. doi: 10.1161/CIRCRESAHA.116.306206, PMID: 27034277 PMC4833475

[12] Sebastian SA Padda I Johal G. Cardiovascular-Kidney-Metabolic (CKM) syndrome: A state-of-the-art review. Curr Probl Cardiol. (2024) 49:102344. doi: 10.1016/j.cpcardiol.2023.102344, PMID: 38103820

[13] Sánchez-García A Rodríguez-Gutiérrez R Mancillas-Adame L González-Nava V Díaz González-Colmenero A Solis RC. Diagnostic accuracy of the triglyceride and glucose index for insulin resistance: A systematic review. Int J Endocrinol. (2020) 2020:4678526. doi: 10.1155/2020/4678526, PMID: 32256572 PMC7085845

[14] Cheng H Huang W Huang X Miao W Huang Y Hu Y. The triglyceride glucose index predicts short-term mortality in non-diabetic patients with acute heart failure. Adv Clin Exp Med. (2024) 33:103–10. doi: 10.17219/acem/166043, PMID: 37326578

[15] Zhou Q Yang J Tang H Guo Z Dong W Wang Y. High triglyceride-glucose (TyG) index is associated with poor prognosis of heart failure with preserved ejection fraction. Cardiovasc Diabetol. (2023) 22:263. doi: 10.1186/s12933-023-02001-4, PMID: 37775762 PMC10541699

[16] Zhou Y Wang C Che H Cheng L Zhu D Rao C. Association between the triglyceride-glucose index and the risk of mortality among patients with chronic heart failure: results from a retrospective cohort study in China. Cardiovasc Diabetol. (2023) 22:171. doi: 10.1186/s12933-023-01895-4, PMID: 37420232 PMC10329381

[17] Han S Wang C Tong F Li Y Li Z Sun Z. Triglyceride glucose index and its combination with the Get with the Guidelines-Heart Failure score in predicting the prognosis in patients with heart failure. Front Nutr. (2022) 9:950338. doi: 10.3389/fnut.2022.950338, PMID: 36159483 PMC9493032

[18] Özcan KS Hayıroğlu MI Çınar T. Admission triglyceride-glucose index is predictor of long-term mortality and appropriate implantable cardiac defibrillator therapy in patients with heart failure. biomark Med. (2023) 17:487–96. doi: 10.2217/bmm-2023-0113, PMID: 37522225

[19] Sun T Huang X Zhang B Ma M Chen Z Zhao Z. Prognostic significance of the triglyceride-glucose index for patients with ischemic heart failure after percutaneous coronary intervention. Front Endocrinol (Lausanne). (2023) 14:1100399. doi: 10.3389/fendo.2023.1100399, PMID: 36814584 PMC9939475

[20] Huang R Wang Z Chen J Bao X Xu N Guo S. Prognostic value of triglyceride glucose (TyG) index in patients with acute decompensated heart failure. Cardiovasc Diabetol. (2022) 21:88. doi: 10.1186/s12933-022-01507-7, PMID: 35641978 PMC9158138

[21] Dhindsa DS Sandesara PB Shapiro MD Wong ND. The evolving understanding and approach to residual cardiovascular risk management. Front Cardiovasc Med. (2020) 7:88. doi: 10.3389/fcvm.2020.00088, PMID: 32478100 PMC7237700

[22] Pammer A Klobučar I Stadler JT Meissl S Habisch H Madl T. Impaired HDL antioxidant and anti-inflammatory functions are linked to increased mortality in acute heart failure patients. Redox Biol. (2024) 76:103341. doi: 10.1016/j.redox.2024.103341, PMID: 39244794 PMC11406013

[23] Gao Q Feng L Zhou W Li X Yin L Wang Y. Non-traditional blood lipid indices for metabolism dysfunction-associated fatty liver disease prediction in non-obese type 2 diabetes mellitus. Diabetes Metab Syndr Obes. (2023) 16:2345–54. doi: 10.2147/DMSO.S418020, PMID: 37577041 PMC10416783

[24] Tong Y Wang Y Chen X Qin B Liu Y Cui Y. The triglyceride glucose: high-density lipoprotein cholesterol ratio is associated with coronary artery calcification evaluated via non-gated chest CT. Cardiovasc Diabetol. (2024) 23:376. doi: 10.1186/s12933-024-02464-z, PMID: 39449019 PMC11515353

[25] He C Cao T Lin Q. Hitachi LABOSPECT008 fully automatic biochemical analyzer performance verification. In: Proceedings of the 2016 Sichuan Medical Doctors Association’s Third Annual Meeting of Laboratory Physicians in the Province (2016). p. 154–4.

[26] Liu Z Wang X Wang J. Sysmex XN-3000 performance verification of automatic blood cell analyzer. Med Equipment. (2019) 32:51–4. doi: 10.3969/j.issn.1002-2376.2019.07.027

[27] Wax Y. Collinearity diagnosis for a relative risk regression analysis: an application to assessment of diet-cancer relationship in epidemiological studies. Stat Med. (1992) 11:1273–87. doi: 10.1002/sim.4780111003, PMID: 1518991

[28] Kuang M Qiu J Li D Hu C Zhang S Sheng G. The newly proposed Metabolic Score for Visceral Fat is a reliable tool for identifying non-alcoholic fatty liver disease, requiring attention to age-specific effects in both sexes. Front Endocrinol (Lausanne). (2023) 14:1281524. doi: 10.3389/fendo.2023.1281524, PMID: 38089634 PMC10711077

[29] Available online at: https://www.wavemetrics.com/products/igorpro/creatinggraphs/3dandvolume/surface (Accessed February 20 2025).

[30] Emdin M Pompella A Paolicchi A. Gamma-glutamyltransferase, atherosclerosis, and cardiovascular disease: triggering oxidative stress within the plaque. Circulation. (2005) 112:2078–80. doi: 10.1161/CIRCULATIONAHA.105.571919, PMID: 16203922

[31] Reina-Couto M Pereira-Terra P Quelhas-Santos J Silva-Pereira C Albino-Teixeira A Sousa T. Inflammation in human heart failure: major mediators and therapeutic targets. Front Physiol. (2021) 12:746494. doi: 10.3389/fphys.2021.746494, PMID: 34707513 PMC8543018

[32] Andrassy RJ Durr ED. Albumin: use in nutrition and support. Nutr Clin Pract. (1988) 3:226–9. doi: 10.1177/0115426588003006226, PMID: 3145397

[33] VanderWeele TJ. Mediation analysis: A practitioner’s guide. Annu Rev Public Health. (2016) 37:17–32. doi: 10.1146/annurev-publhealth-032315-021402, PMID: 26653405

[34] Potočnjak I Degoricija V Trbušić M Pregartner G Berghold A Marsche G. Serum concentration of HDL particles predicts mortality in acute heart failure patients. Sci Rep. (2017) 7:46642. doi: 10.1038/srep46642, PMID: 28418031 PMC5394530

[35] Potočnjak I Degoricija V Trbušić M Terešak SD Radulović B Pregartner G. Metrics of high-density lipoprotein function and hospital mortality in acute heart failure patients. PloS One. (2016) 11:e0157507. doi: 10.1371/journal.pone.0157507, PMID: 27304214 PMC4909230

[36] Bettencourt P Azevedo A Pimenta J Friões F Ferreira S Ferreira A. N-terminal-pro-brain natriuretic peptide predicts outcome after hospital discharge in heart failure patients. Circulation. (2004) 110:2168–74. doi: 10.1161/01.CIR.0000144310.04433.BE, PMID: 15451800

[37] O’Brien RJ Squire IB Demme B Davies JE Ng LL. Pre-discharge, but not admission, levels of NT-proBNP predict adverse prognosis following acute LVF. Eur J Heart Fail. (2003) 5:499–506. doi: 10.1016/s1388-9842(03)00098-9, PMID: 12921811

[38] Bayés-Genís A Lopez L Zapico E Cotes C Santaló M Ordonez-Llanos J. NT-ProBNP reduction percentage during admission for acutely decompensated heart failure predicts long-term cardiovascular mortality. J Card Fail. (2005) 11:S3–8. doi: 10.1016/j.cardfail.2005.04.006, PMID: 15948093

[39] Bayes-Genis A Aimo A Jhund P Richards M de Boer RA Arfsten H. Biomarkers in heart failure clinical trials. A review from the Biomarkers Working Group of the Heart Failure Association of the European Society of Cardiology. Eur J Heart Fail. (2022) 24:1767–77. doi: 10.1002/ejhf.2675, PMID: 36073112

[40] Núñez J de la Espriella R Rossignol P Voors AA Mullens W Metra M. Congestion in heart failure: a circulating biomarker-based perspective. A review from the Biomarkers Working Group of the Heart Failure Association, European Society of Cardiology. Eur J Heart Fail. (2022) 24:1751–66. doi: 10.1002/ejhf.2664, PMID: 36039656

[41] Metra M Cotter G Davison BA Felker GM Filippatos G Greenberg BH. Effect of serelaxin on cardiac, renal, and hepatic biomarkers in the Relaxin in Acute Heart Failure (RELAX-AHF) development program: correlation with outcomes. J Am Coll Cardiol. (2013) 61:196–206. doi: 10.1016/j.jacc.2012.11.005, PMID: 23273292

[42] Akirov A Masri-Iraqi H Atamna A Shimon I. Low albumin levels are associated with mortality risk in hospitalized patients. Am J Med. (2017) 130:1465.e11–1465.e19. doi: 10.1016/j.amjmed.2017.07.020, PMID: 28803138

[43] Grimm G Haslacher H Kampitsch T Endler G Marsik C Schickbauer T. Sex differences in the association between albumin and all-cause and vascular mortality. Eur J Clin Invest. (2009) 39:860–5. doi: 10.1111/j.1365-2362.2009.02189.x, PMID: 19645741

[44] Shannon CM Ballew SH Daya N Zhou L Chang AR Sang Y. Serum albumin and risks of hospitalization and death: Findings from the Atherosclerosis Risk in Communities study. J Am Geriatr Soc. (2021) 69:2865–76. doi: 10.1111/jgs.17313, PMID: 34298583 PMC8582595

[45] Kabat GC Kim MY Manson JE Lessin L Lin J Wassertheil-Smoller S. White blood cell count and total and cause-specific mortality in the women’s health initiative. Am J Epidemiol. (2017) 186:63–72. doi: 10.1093/aje/kww226, PMID: 28369251 PMC5860271

[46] Rimmer E Garland A Kumar A Doucette S Houston BL Menard CE. White blood cell count trajectory and mortality in septic shock: a historical cohort study. Can J Anaesth. (2022) 69:1230–9. doi: 10.1007/s12630-022-02282-5, PMID: 35902458 PMC9334545

[47] Im KM Kim EY. Reducing in-hospital and 60-day mortality in critically ill patients after surgery with strict nutritional supplementation: A prospective, single-labeled, randomized controlled trial. Nutrients. (2023) 15:4684. doi: 10.3390/nu15214684, PMID: 37960337 PMC10648808

[48] Yamanashi T Sullivan EJ Comp KR Nishizawa Y Akers CC Chang G. Anti-inflammatory medication use associated with reduced delirium risk and all-cause mortality: A retrospective cohort study. J Psychosom Res. (2023) 168:111212. doi: 10.1016/j.jpsychores.2023.111212, PMID: 36963165

[49] Yu X Chen Q Xu Lou I. Dietary strategies and nutritional supplements in the management of heart failure: a systematic review. Front Nutr. (2024) 11:1428010. doi: 10.3389/fnut.2024.1428010, PMID: 39464682 PMC11502353

[50] Kida K Miyajima I Suzuki N Greenberg BH Akashi YJ. Nutritional management of heart failure. J Cardiol. (2023) 81:283–91. doi: 10.1016/j.jjcc.2022.11.001, PMID: 36370995

[51] Van Tassell BW Canada J Carbone S Trankle C Buckley L Oddi Erdle C. Interleukin-1 blockade in recently decompensated systolic heart failure: results from REDHART (Recently decompensated heart failure Anakinra response trial). Circ Heart Fail. (2017) 10:e004373. doi: 10.1161/CIRCHEARTFAILURE.117.004373, PMID: 29141858 PMC5699505

[52] Cotter G Davison BA Freund Y Voors AA Edwards C Novosadova M. Burst steroid therapy for acute heart failure: The CORTAHF randomized, open-label, pilot trial. Eur J Heart Fail. (2024) 26:2282–92. doi: 10.1002/ejhf.3452, PMID: 39211989

[53] Pascual-Figal D Núñez J Pérez-Martínez MT González-Juanatey JR Taibo-Urquia M Llàcer-Iborra P. Colchicine in acutely decompensated heart failure: the COLICA trial. Eur Heart J. (2024) 45:4826–36. doi: 10.1093/eurheartj/ehae538, PMID: 39211951 PMC11637759

[54] Davison BA Abbate A Cotter G Pascual-Figal D Van Tassell B Villota JN. Effects of anti-inflammatory therapy in acute heart failure: a systematic review and meta-analysis. Heart Fail Rev. (2025) 30:575–87. doi: 10.1007/s10741-025-10491-5, PMID: 39939545

[55] Huang Y Zhou Y Xu Y Wang X Zhou Z Wu K. Inflammatory markers link triglyceride-glucose index and obesity indicators with adverse cardiovascular events in patients with hypertension: insights from three cohorts. Cardiovasc Diabetol. (2025) 24:11. doi: 10.1186/s12933-024-02571-x, PMID: 39780176 PMC11716003

[56] Zhu H Chen Y Ding D Chen H. Association between different insulin resistance indices and all-cause mortality in patients with diabetic kidney disease: a prospective cohort study. Front Endocrinol (Lausanne). (2025) 15:1427727. doi: 10.3389/fendo.2024.1427727, PMID: 39872311 PMC11769815

[57] Xu H Xie J Xia Y Niu H Wang H Zhan F. Association of TyG index with mortality at 28 days in sepsis patients in intensive care from MIMIC IV database. Sci Rep. (2025) 15:2344. doi: 10.1038/s41598-025-86746-w, PMID: 39833386 PMC11747252

[58] Zhang HJ Han LL Luo W Hu M Zhang HZ Liao YL. The triglyceride-glucose index: a predictor of mortality risk among myocardial infarction survivors. Sci Rep. (2024) 14:27512. doi: 10.1038/s41598-024-78056-4, PMID: 39528543 PMC11554645

[59] Liu C Liang D Xiao K Xie L. Association between the triglyceride-glucose index and all-cause and CVD mortality in the young population with diabetes. Cardiovasc Diabetol. (2024) 23:171. doi: 10.1186/s12933-024-02269-0, PMID: 38755682 PMC11097545

[60] Hao B Lyu L Xu J Zhu X Xu C Gao W. The relationship between triglyceride-glucose index and prospective key clinical outcomes in patients hospitalised for coronary artery disease. Cardiovasc Diabetol. (2024) 23:40. doi: 10.1186/s12933-024-02132-2, PMID: 38254088 PMC10804527

[61] Zhang Q Xiao S Jiao X Shen Y. The triglyceride-glucose index is a predictor for cardiovascular and all-cause mortality in CVD patients with diabetes or pre-diabetes: evidence from NHANES 2001-2018. Cardiovasc Diabetol. (2023) 22:279. doi: 10.1186/s12933-023-02030-z, PMID: 37848879 PMC10583314

[62] Zhang H Wang L Zhang Q Song Y Cai M Bao J. Non-linear association of triglyceride-glucose index with cardiovascular and all-cause mortality in T2DM patients with diabetic kidney disease: NHANES 2001-2018 retrospective cohort study. Lipids Health Dis. (2024) 23:253. doi: 10.1186/s12944-024-02249-z, PMID: 39154178 PMC11330591

[63] Zhao M Xiao M Tan Q Lu F. Triglyceride glucose index as a predictor of mortality in middle-aged and elderly patients with type 2 diabetes in the US. Sci Rep. (2023) 13:16478. doi: 10.1038/s41598-023-43512-0, PMID: 37777574 PMC10542790

[64] Yu Y Wang J Ding L Huang H Cheng S Deng Y. Sex differences in the nonlinear association of triglyceride glucose index with all-cause and cardiovascular mortality in the general population. Diabetol Metab Syndr. (2023) 15:136. doi: 10.1186/s13098-023-01117-7, PMID: 37349808 PMC10288670

[65] Li G Zhong S Wang X Zhuge F. Association of hypoglycaemia with the risks of arrhythmia and mortality in individuals with diabetes - a systematic review and meta-analysis. Front Endocrinol (Lausanne). (2023) 14:1222409. doi: 10.3389/fendo.2023.1222409, PMID: 37645418 PMC10461564

[66] Wright RJ Frier BM. Vascular disease and diabetes: is hypoglycaemia an aggravating factor? Diabetes Metab Res Rev. (2008) 24:353–63. doi: 10.1002/dmrr.865, PMID: 18461635

[67] Benkhoff M Polzin A. Lipoprotection in cardiovascular diseases. Pharmacol Ther. (2024) 264:108747. doi: 10.1016/j.pharmthera.2024.108747, PMID: 39491757

[68] Mineo C Shaul PW. Novel biological functions of high-density lipoprotein cholesterol. Circ Res. (2012) 111:1079–90. doi: 10.1161/CIRCRESAHA.111.258673, PMID: 23023510 PMC3500606

[69] Lüscher TF Landmesser U von Eckardstein A Fogelman AM. High-density lipoprotein: vascular protective effects, dysfunction, and potential as therapeutic target. Circ Res. (2014) 114:171–82. doi: 10.1161/CIRCRESAHA.114.300935, PMID: 24385510

[70] Lui DTW Li L Liu X Xiong X Tang EHM Lee CH. The association of HDL-cholesterol levels with incident major adverse cardiovascular events and mortality in 0.6 million individuals with type 2 diabetes: a population-based retrospective cohort study. BMC Med. (2024) 22:586. doi: 10.1186/s12916-024-03810-4, PMID: 39696353 PMC11657474

[71] Hirata A Sugiyama D Watanabe M Tamakoshi A Iso H Kotani K. Association of extremely high levels of high-density lipoprotein cholesterol with cardiovascular mortality in a pooled analysis of 9 cohort studies including 43,407 individuals: The EPOCH-JAPAN study. J Clin Lipidol. (2018) 12:674–684.e5. doi: 10.1016/j.jacl.2018.01.014, PMID: 29506864

[72] Liu C Dhindsa D Almuwaqqat Z Sun YV Quyyumi AA. Very high high-density lipoprotein cholesterol levels and cardiovascular mortality. Am J Cardiol. (2022) 167:43–53. doi: 10.1016/j.amjcard.2021.11.041, PMID: 35039162

[73] Zhang G Guo J Jin H Wei X Zhu X Jia W. Association between extremely high-density lipoprotein cholesterol and adverse cardiovascular outcomes: a systematic review and meta-analysis. Front Cardiovasc Med. (2023) 10:1201107. doi: 10.3389/fcvm.2023.1201107, PMID: 37441703 PMC10333521

[74] Dron JS Wang J Low-Kam C Khetarpal SA Robinson JF McIntyre AD. Polygenic determinants in extremes of high-density lipoprotein cholesterol. J Lipid Res. (2017) 58:2162–70. doi: 10.1194/jlr.M079822, PMID: 28870971 PMC5665671

[75] Madsen CM Varbo A Nordestgaard BG. Novel insights from human studies on the role of high-density lipoprotein in mortality and noncardiovascular disease. Arterioscler Thromb Vasc Biol. (2021) 41:128–40. doi: 10.1161/ATVBAHA.120.314050, PMID: 33232200

[76] Strange G Playford D Scalia GM Celermajer DS Prior D Codde J. Change in ejection fraction and long-term mortality in adults referred for echocardiography. Eur J Heart Fail. (2021) 23:555–63. doi: 10.1002/ejhf.2161, PMID: 33768605

[77] Wehner GJ Jing L Haggerty CM Suever JD Leader JB Hartzel DN. Routinely reported ejection fraction and mortality in clinical practice: where does the nadir of risk lie? Eur Heart J. (2020) 41:1249–57. doi: 10.1093/eurheartj/ehz550, PMID: 31386109 PMC8204658

[78] Stewart S Playford D Scalia GM Currie P Celermajer DS Prior D. Ejection fraction and mortality: a nationwide register-based cohort study of 499 153 women and men. Eur J Heart Fail. (2021) 23:406–16. doi: 10.1002/ejhf.2047, PMID: 33150657

[79] Ilonze OJ Parsly Read-Button L Cogswell R Hackman A Breathett K Saltzman E. Controversies and conundrums in cardiac cachexia: key questions about wasting in patients with HFrEF. JACC Heart Fail. (2024) 12:1645–60. doi: 10.1016/j.jchf.2024.03.003, PMID: 38727650

[80] Wood N Straw S Cheng CW Hirata Y Pereira MG Gallagher H. Sodium-glucose cotransporter 2 inhibitors influence skeletal muscle pathology in patients with heart failure and reduced ejection fraction. Eur J Heart Fail. (2024) 26:925–35. doi: 10.1002/ejhf.3192, PMID: 38468429

[81] Sullivan MJ Green HJ Cobb FR. Skeletal muscle biochemistry and histology in ambulatory patients with long-term heart failure. Circulation. (1990) 81:518–27. doi: 10.1161/01.cir.81.2.518, PMID: 2297859

[82] Feingold KR Grunfeld C. Effect of inflammation on HDL structure and function. Curr Opin Lipidol. (2016) 27:521–30. doi: 10.1097/MOL.0000000000000333, PMID: 27495134

[83] Marsche G Saemann MD Heinemann A Holzer M. Inflammation alters HDL composition and function: implications for HDL-raising therapies. Pharmacol Ther. (2013) 137:341–51. doi: 10.1016/j.pharmthera.2012.12.001, PMID: 23246719

[84] Putnam K Shoemaker R Yiannikouris F Cassis LA. The renin-angiotensin system: a target of and contributor to dyslipidemias, altered glucose homeostasis, and hypertension of the metabolic syndrome. Am J Physiol Heart Circ Physiol. (2012) 302:H1219–30. doi: 10.1152/ajpheart.00796.2011, PMID: 22227126 PMC3311482

