# Supplementary material for: Independent and combined associations of high-density lipoprotein cholesterol-modified triglyceride-glucose index with all-cause and cardiovascular mortality in patients with acute decompensated heart failure
Source: Front Endocrinol (Lausanne). 2025 Jul 29;16:1629066. doi: 10.3389/fendo.2025.1629066 (PMC12339345; doi:10.3389/fendo.2025.1629066)
Supplement: Supplementary file 1 [file Table1.docx]

Supplementary Table 1: Collinearity diagnostics steps.

|  | VIF | | |
| --- | --- | --- | --- |
|  | Step 1 | Step 2 | Step 3 |
| TyG/HDL-C ratio | 3.1 | 3.1 | 1.6 |
| Gender | 1.3 | 1.3 | 1.3 |
| Age | 1.5 | 1.5 | 1.5 |
| Hypertension | 1.3 | 1.3 | 1.3 |
| Diabetes | 1.4 | 1.4 | 1.4 |
| Stroke | 1.1 | 1.1 | 1.1 |
| CHD | 1.2 | 1.2 | 1.2 |
| NYHA classification | 1.1 | 1.1 | 1.1 |
| SBP | 1.8 | 1.8 | 1.8 |
| DBP | 1.6 | 1.6 | 1.6 |
| Drinking status | 1.5 | 1.5 | 1.5 |
| Smoking status | 1.6 | 1.6 | 1.6 |
| LVEF | 1.3 | 1.3 | 1.3 |
| WBC | 1.4 | 1.4 | 1.4 |
| RBC | 1.5 | 1.5 | 1.5 |
| PLT | 1.2 | 1.2 | 1.2 |
| Alb | 1.3 | 1.3 | 1.3 |
| ALT | 7.7 | NA | NA |
| AST | 7.3 | 1.1 | 1.1 |
| GGT | 1.2 | 1.1 | 1.1 |
| Cr | 2.5 | 2.5 | 2.5 |
| BUN | 2.9 | 2.9 | 2.9 |
| UA | 1.6 | 1.6 | 1.6 |
| TG | 1.5 | 1.5 | 1.5 |
| TC | 1.8 | 1.8 | 1.5 |
| HDL-C | 3.4 | 3.4 | NA |
| LDL-C | 1 | 1 | 1 |
| FPG | 1.5 | 1.5 | 1.5 |
| NT-proBNP | 1.3 | 1.3 | 1.3 |

VIF: variance inflation factor; VIF = 1/(1-R^2^). Abbreviations as in Table ​1.

Note: The variables with VIF>5 will be regarded as collinear variables.

Supplementary Table 2: Using all-cause mortality as the dependent variable, the proportional hazards assumption for covariates included in the model was tested via Schoenfeld residuals.

| Variable | chisq | df | *P* value |
| --- | --- | --- | --- |
| gender | 1.694 | 1 | 0.193 |
| age | 3.028 | 1 | 0.082 |
| Hypertension | 0.39 | 1 | 0.532 |
| Diabetes | 1.787 | 1 | 0.181 |
| Stroke | 0.003 | 1 | 0.956 |
| CHD | 0.815 | 1 | 0.367 |
| NYHA classification | 0.09 | 1 | 0.764 |
| Drinking status | 0.378 | 1 | 0.539 |
| Smoking status | 0.001 | 1 | 0.98 |
| LVEF | 3.178 | 1 | 0.075 |
| WBC | 0.208 | 1 | 0.648 |
| RBC | 0.986 | 1 | 0.321 |
| PLT | 7.077 | 1 | 0.008 |
| Alb | 0 | 1 | 0.991 |
| GGT | 0.076 | 1 | 0.782 |
| Cr | 0.137 | 1 | 0.712 |
| BUN | 0.474 | 1 | 0.491 |
| UA | 0.149 | 1 | 0.699 |
| TC | 0.298 | 1 | 0.585 |
| LDL | 0.468 | 1 | 0.494 |
| NT-proBNP | 0.135 | 1 | 0.713 |
| AST | 4.734 | 1 | 0.03 |
| TyG/HDL-C ratio | 0.03 | 1 | 0.863 |
| Global Schoenfeld Test p | 34.941 | 23 | 0.053 |

Abbreviations as in Table ​1.

Supplementary Table 3: Using Cardiovascular mortality as the dependent variable, the proportional hazards assumption for covariates included in the model was tested via Schoenfeld residuals.

| Variable | chisq | df | p.value |
| --- | --- | --- | --- |
| gender | 1.613 | 1 | 0.204 |
| age | 2.036 | 1 | 0.154 |
| Hypertension | 0.018 | 1 | 0.894 |
| Diabetes | 0.26 | 1 | 0.61 |
| Stroke | 0.382 | 1 | 0.536 |
| CHD | 0.236 | 1 | 0.627 |
| NYHA classification | 0.201 | 1 | 0.654 |
| Drinking status | 0.188 | 1 | 0.665 |
| Smoking status | 0.011 | 1 | 0.918 |
| LVEF | 1.738 | 1 | 0.187 |
| WBC | 0.015 | 1 | 0.902 |
| RBC | 0.204 | 1 | 0.652 |
| PLT | 4.998 | 1 | 0.025 |
| Alb | 0.023 | 1 | 0.879 |
| GGT | 0.182 | 1 | 0.669 |
| Cr | 0.077 | 1 | 0.781 |
| BUN | 0.002 | 1 | 0.963 |
| UA | 0.003 | 1 | 0.96 |
| TC | 0 | 1 | 1 |
| LDL | 0.176 | 1 | 0.675 |
| NT-proBNP | 0.051 | 1 | 0.821 |
| AST | 1.217 | 1 | 0.27 |
| TyG/HDL-C ratio | 0.097 | 1 | 0.755 |
| Global Schoenfeld Test p | 15.955 | 23 | 0.857 |

Abbreviations as in Table ​1.

Supplementary Table 4. The missing number and rate of covariates.

|  | Non- Missing | Missing |
| --- | --- | --- |
| Gender | 2329 | 0 |
| Age | 2329 | 0 |
| Hypertension | 2329 | 0 |
| Diabetes | 2329 | 0 |
| Stroke | 2329 | 0 |
| CHD | 2329 | 0 |
| NYHA classification | 2329 | 0 |
| SBP | 2329 | 0 |
| DBP | 2329 | 0 |
| Drinking status | 2329 | 0 |
| Smoking status | 2329 | 0 |
| LVEF | 2225 | 104 |
| WBC | 2308 | 21 |
| RBC | 2308 | 21 |
| PLT | 2308 | 21 |
| Alb | 2311 | 18 |
| ALT | 2311 | 18 |
| AST | 2313 | 16 |
| GGT | 2311 | 18 |
| Cr | 2303 | 26 |
| BUN | 2303 | 26 |
| UA | 2302 | 27 |
| TG | 2329 | 0 |
| TC | 2329 | 0 |
| HDL-C | 2329 | 0 |
| LDL-C | 2329 | 0 |
| FPG | 2329 | 0 |
| NT-proBNP | 2329 | 0 |
| TyG/HDL-C ratio | 2329 | 0 |

Abbreviations as in Table 1.

Supplementary Table 5: Multivariate Cox regression analysis of the association between the TyG/HDL-C ratio and non-cardiovascular mortality in ADHF patients.

|  | HR (95%CI) P-value |
| --- | --- |
| TyG/HDL-C ratio (Per SD increase) | 1.26 (0.99, 1.61) 0.0651 |
| TyG/HDL-C ratio (quartiles) |  |
| Q1 | 1.0 |
| Q2 | 1.32 (0.35, 5.04) 0.6855 |
| Q3 | 2.40 (0.73, 7.88) 0.1501 |
| Q4 | 4.26 (1.35, 13.45) 0.0135 |
| *P*-trend | 0.0081 |

Adjusted for gender, age, drinking status, smoking status, hypertension, diabetes, stroke, CHD, NYHA classification, LVEF, WBC, RBC, PLT, Alb, AST, GGT, Cr, BUN, UA, TC, LDL-C, NT-proBNP.

Supplementary Table 6: Evaluating the incremental prognostic value of adding the TyG/HDL-C ratio to the NT-proBNP model for predicting 30-day mortality.

| Model | C-index | *P* value | NRI (95% CI) | *P* value |
| --- | --- | --- | --- | --- |
| All-cause mortality |  |  |  |  |
| NT-proBNP Model | 0.67 |  | Ref |  |
| + TyG/HDL-C ratio | 0.72 | <0.01 | 0.19 (0.07, 0.27) | 0.01 |
| Cardiovascular mortality |  |  |  |  |
| NT-proBNP Model | 0.71 |  |  |  |
| + TyG/HDL-C ratio | 0.75 | <0.01 | 0.15 (0.01, 0.26) | 0.04 |

CI confidence interval; NRI net reclassification improvement. other abbreviations can be seen in Table 1.

Supplementary Table 7: Mediated analysis was performed to explore the roles of inflammation, oxidative stress and nutritional pathways in the association between TyG/HDL and the 30-day mortality rate in ADHF patients.

| Mediator | Total effect | Mediation effect | Direct effect | PM(%) | *P*-value of PM |
| --- | --- | --- | --- | --- | --- |
| All-cause mortality |  |  |  |  |  |
| WBC | 0.016 (0.006, 0.025) | 0.002 (0.000, 0.003) | 0.014 (0.004, 0.024) | 9.59 | 0.004 |
| GGT | 0.016 (0.006, 0.025) | -0.000 (-0.000, 0.000) | 0.016 (0.007, 0.025) | 1.95 | 0.376 |
| ALB | 0.016 (0.006, 0.025) | 0.004 (0.002, 0.006) | 0.012 (0.003, 0.020) | 26.51 | <0.001 |
| Cardiovascular mortality |  |  |  |  |  |
| WBC | 0.009 (0.001, 0.016) | 0.001 (0.000, 0.013) | 0.007 (-0.001, 0.014) | 14.04 | 0.024 |
| GGT | 0.009 (0.001, 0.016) | 0.000 (-0.000, 0.000) | 0.009 (0.002, 0.015) | 0.66 | 0.914 |
| ALB | 0.009 (0.001, 0.016) | 0.002 (0.001, 0.004) | 0.007 (0.001, 0.014) | 21.40 | 0.066 |

Abbreviations: PM: proportion mediate; ADHF: acute decompensated heart failure; other abbreviations as in Table ​1.

Note: Model adjusted for the same covariates as in model III (Table 2), except for the mediator variable.
