# Supplementary material for: Independent and combined associations of high-density lipoprotein cholesterol-modified triglyceride-glucose index with all-cause and cardiovascular mortality in patients with acute decompensated heart failure
Source: Front Endocrinol (Lausanne). 2025 Jul 29;16:1629066. doi: 10.3389/fendo.2025.1629066 (PMC12339345; doi:10.3389/fendo.2025.1629066)
Supplement: Supplementary file 2 [file DataSheet1.docx]

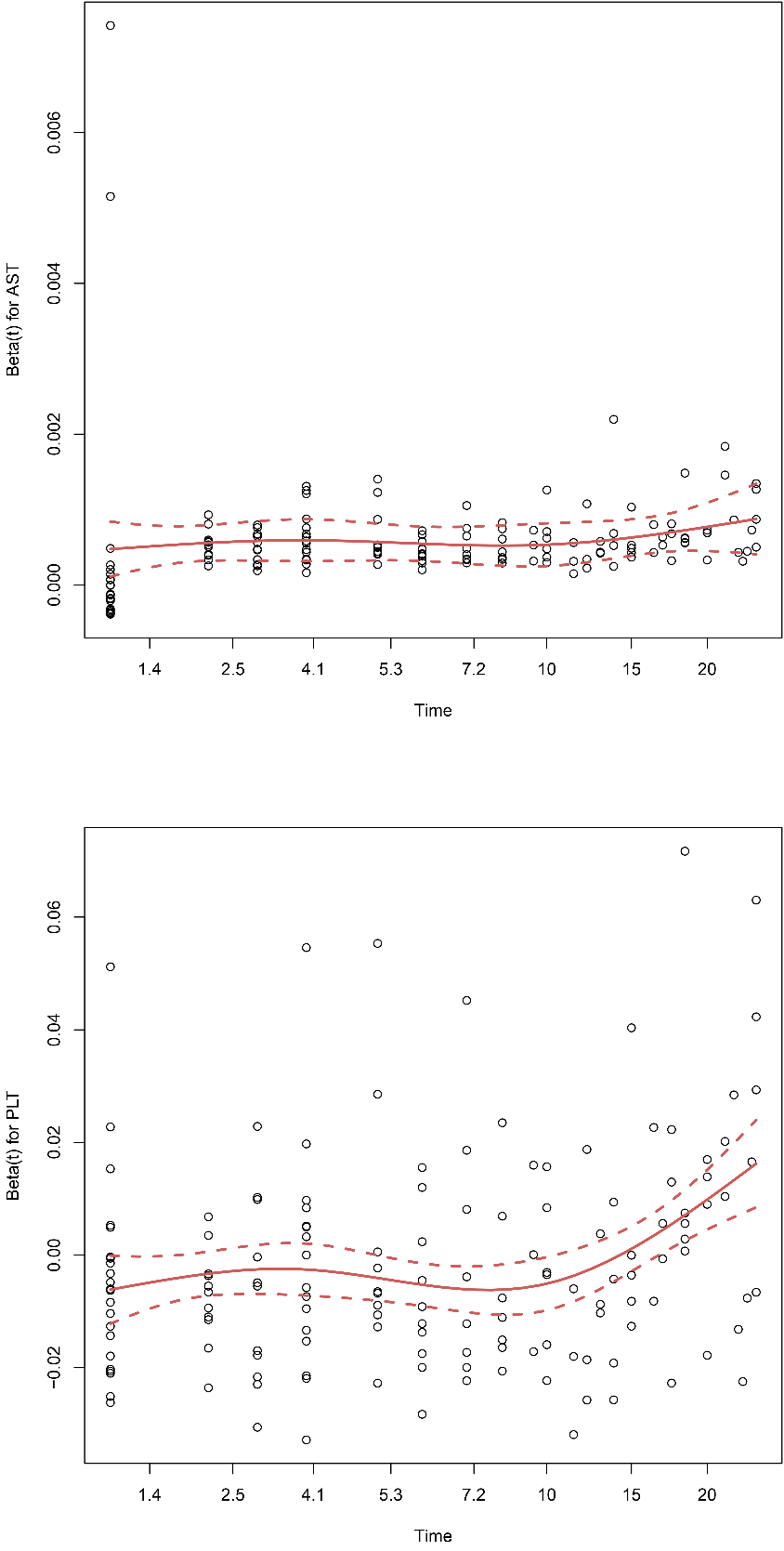


**Supplementary Figure 1:** Schoenfeld residual plot of AST and PLT over time with 30-day mortality in ADHF patients as the dependent variable.


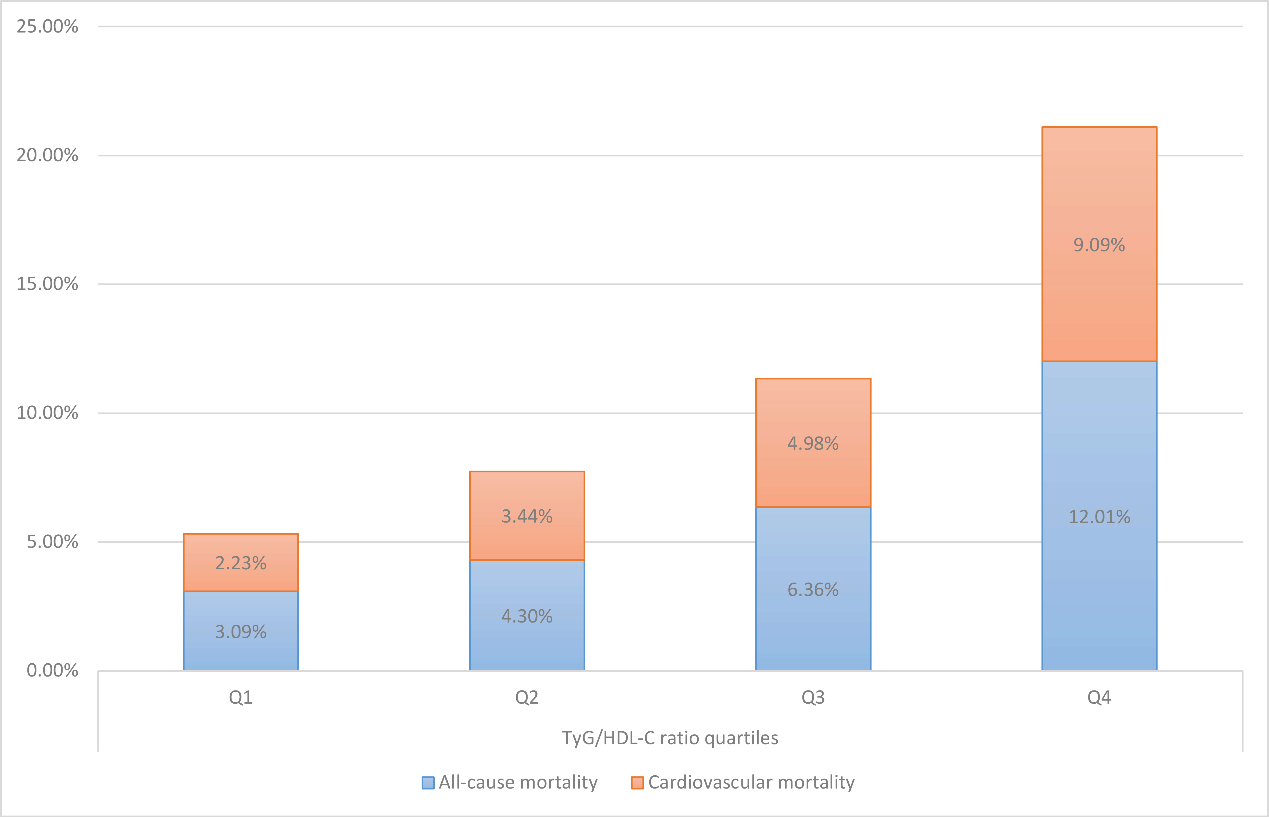


**Supplementary Figure 2:** Stacked bar chart depicting 30-day all-cause and cardiovascular mortality among ADHF patients stratified by TyG/HDL-C ratio quartiles. TyG/HDL-C ratio: triglyceride-glucose index/high-density lipoprotein cholesterol ratio; ADHF: acute decompensated heart failure.


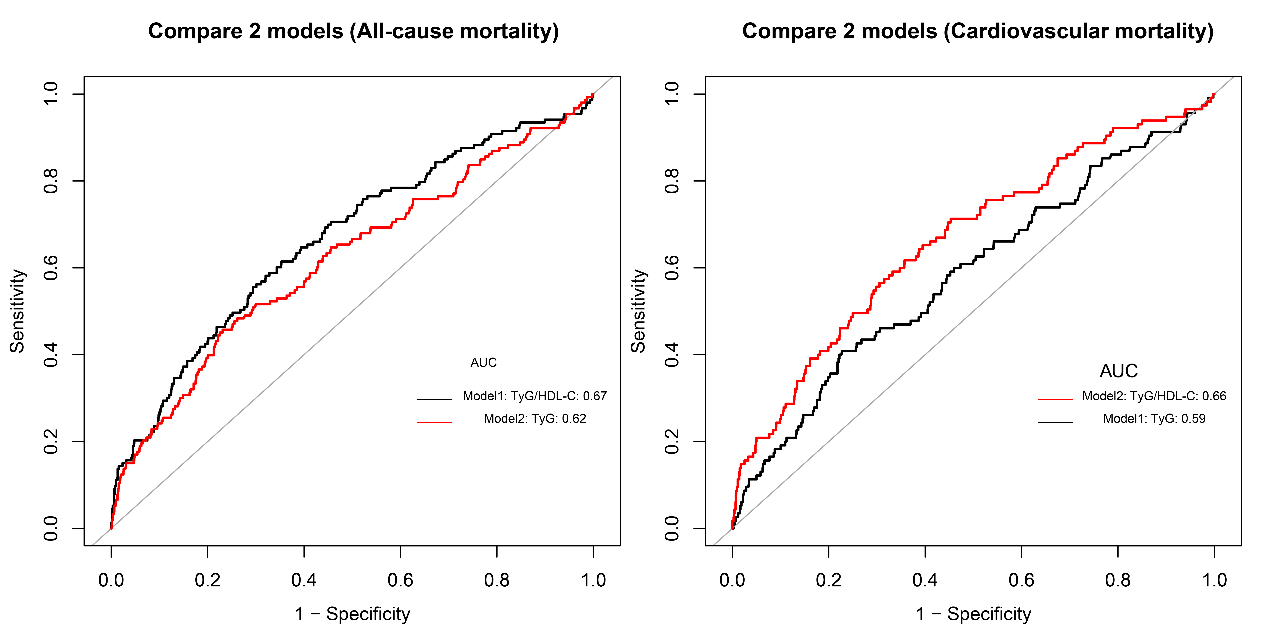


**Supplementary Figure 3:** Receiver operating characteristic curve analysis evaluating the predictive performance of the TyG index and TyG/HDL-C ratio for cardiovascular and all-cause mortality in patients with ADHF. TyG/HDL-C ratio: triglyceride-glucose index/high-density lipoprotein cholesterol ratio; ADHF: acute decompensated heart failure.


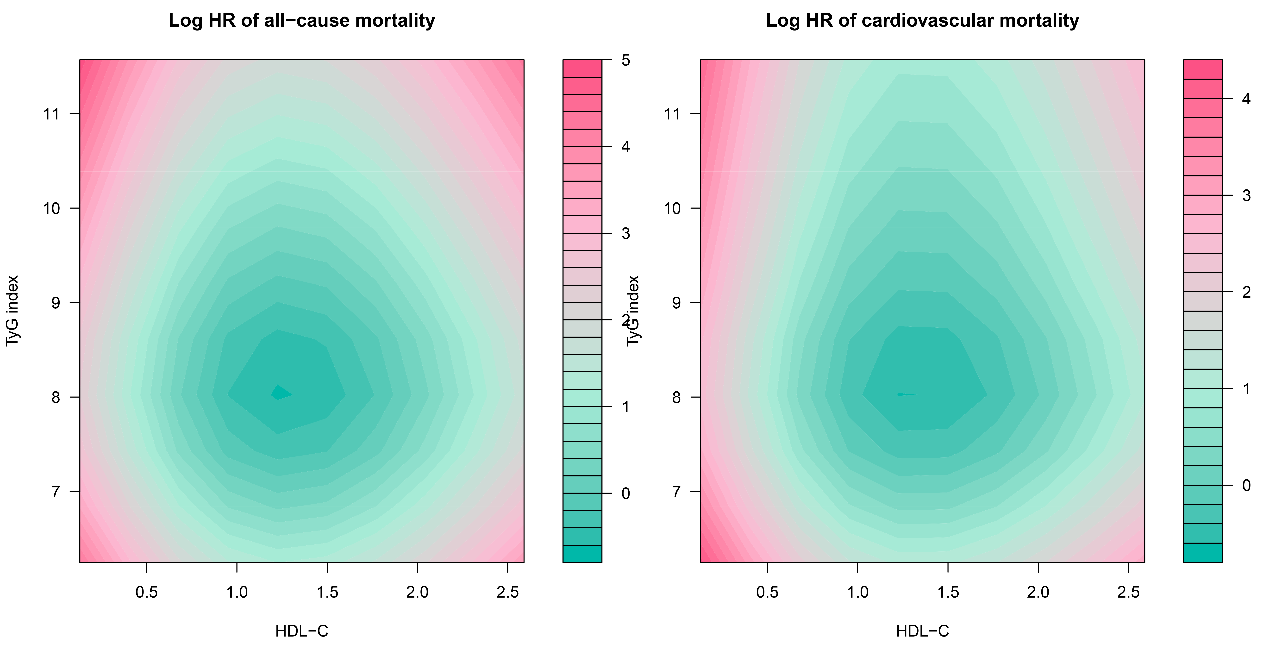


**Supplementary Figure 4**: Heatmap of potential interaction effect between TyG index and HDL-C on 30-day mortality in ADHF patients. The color band represents the effect of TyG index or CRP level on the probability of 30-day mortality. The deep the red color, the stronger the positive correlation; the deep the cyan color, the stronger the negative correlation.
